# Direct Transcriptional Consequences of Somatic Mutation in Breast Cancer

**DOI:** 10.1016/j.celrep.2016.07.028

**Published:** 2016-08-04

**Authors:** Adam Shlien, Keiran Raine, Fabio Fuligni, Roland Arnold, Serena Nik-Zainal, Serge Dronov, Lira Mamanova, Andrej Rosic, Young Seok Ju, Susanna L. Cooke, Manasa Ramakrishna, Elli Papaemmanuil, Helen R. Davies, Patrick S. Tarpey, Peter Van Loo, David C. Wedge, David R. Jones, Sancha Martin, John Marshall, Elizabeth Anderson, Claire Hardy, ICGC Breast Cancer Working Group, Oslo Breast Cancer Research Consortium, Violetta Barbashina, Samuel A.J.R. Aparicio, Torill Sauer, Øystein Garred, Anne Vincent-Salomon, Odette Mariani, Sandrine Boyault, Aquila Fatima, Anita Langerød, Åke Borg, Gilles Thomas, Andrea L. Richardson, Anne-Lise Børresen-Dale, Kornelia Polyak, Michael R. Stratton, Peter J. Campbell

**Affiliations:** 1 Cancer Genome Project, Wellcome Trust Sanger Institute, Hinxton, Cambridgeshire CB10 1SA, UK; 2 Department of Human Genetics, University of Leuven, 3000 Leuven, Belgium; 3 Breakthrough Breast Cancer, The Institute of Cancer Research, London SM2 5NG, UK; 4 British Columbia Cancer Agency, Vancouver, BC V5Z 1L3, Canada; 5 Department of Pathology, Oslo University Hospital, 0450 Oslo, Norway; 6 Institut Curie, 75005 Paris, France; 7 Synergie Lyon Cancer, Centre Léon Bérard, 69008 Lyon, France; 8 Dana-Farber Cancer Institute, Boston, MA 02215, USA; 9 Department of Genetics, Institute for Cancer Research, Oslo University Hospital Radiumhospitalet, 0379 Oslo, Norway; 10 K.G. Jebsen Center for Breast Cancer Research, Institute for Clinical Medicine, Faculty of Medicine, University of Oslo, 0316 Oslo, Norway; 11 Department of Oncology, Lund University, SE-221 00 Lund, Sweden; 12 Department of Medical Oncology, Dana-Farber Cancer Institute, Boston, MA 02215, USA; 13 Department of Haematology, Addenbrooke’s Hospital, Cambridge CB2 0QQ, UK; 14 Department of Haematology, University of Cambridge, Cambridge CB2 1TN, UK; 15 Department of Genetics and Genome Biology, The Hospital for Sick Children, Toronto, ON M5G 1X8, Canada

## Abstract

Disordered transcriptomes of cancer encompass direct effects of somatic mutation on transcription, coordinated secondary pathway alterations, and increased transcriptional noise. To catalog the rules governing how somatic mutation exerts direct transcriptional effects, we developed an exhaustive pipeline for analyzing RNA sequencing data, which we integrated with whole genomes from 23 breast cancers. Using X-inactivation analyses, we found that cancer cells are more transcriptionally active than intermixed stromal cells. This is especially true in estrogen receptor (ER)-negative tumors. Overall, 59% of substitutions were expressed. Nonsense mutations showed lower expression levels than expected, with patterns characteristic of nonsense-mediated decay. 14% of 4,234 rearrangements caused transcriptional abnormalities, including exon skips, exon reusage, fusions, and premature polyadenylation. We found productive, stable transcription from sense-to-antisense gene fusions and gene-to-intergenic rearrangements, suggesting that these mutation classes drive more transcriptional disruption than previously suspected. Systematic integration of transcriptome with genome data reveals the rules by which transcriptional machinery interprets somatic mutation.

## Highlights

• Greater transcriptional activity in cancer than stromal cells, particularly when ER-ve
• Intron mutations only infrequently affect splicing, even at essential splice sites
• Distinctive RNA effects of sense-to-antisense and gene-to-intergenic rearrangements
• Exhaustive pipeline for identifying aberrant transcripts from RNA-sequencing data

## Graphical Abstract

**Figure:**
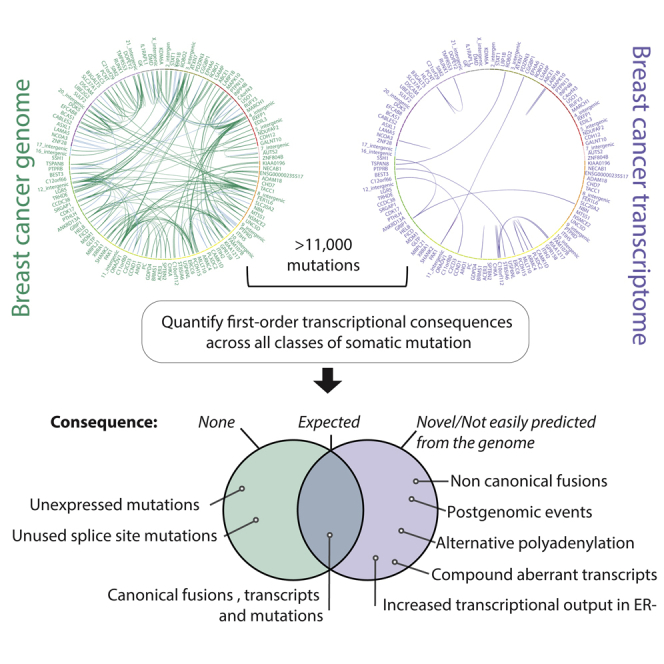

## Teaser

Shlien et al. quantify first-order transcriptional consequences across all classes of somatic mutation in well-annotated breast cancers. They find that cancer cells are more transcriptionally active than stromal cells, especially in ER-negative cancers. Rearrangements that break genes or place them in opposite orientation are an unexpectedly potent source of fusions.

## Introduction

Somatic mutation underpins the development of cancer, and most solid tumors have thousands to tens of thousands of point mutations, coupled with tens to hundreds of genomic rearrangements and copy-number changes (Garraway and Lander, 2013, Stratton et al., 2009). Small numbers of these, known as driver mutations, dysregulate the fundamental cellular processes involved in normal tissue homeostasis, and they confer a selective advantage to the clone. A critical point is that Darwinian selection acts on *phenotype*, and so, for a somatic mutation to drive cancer, it must manifest a phenotypic effect. Transcription is the primary conduit by which changes in the genomic code are translated into cellular phenotype, with the corollary that it is a necessary criterion of driver mutations that they directly induce a change in transcript structure. Altered transcript structure can take many forms, including the creation of fusion genes by genomic rearrangement, interference with RNA splicing at mutated splice sites, alteration of the codon sequence for missense substitutions, and over- or under-expression of genes through copy-number alterations or mutation in regulatory regions.

Beyond the primary and direct effects of somatic mutation on transcript structure, there may be a series of downstream, secondary alterations in the transcriptome occurring as a consequence of the primary effect. Most studies of the transcriptome in cancer, including those from large-scale efforts such as The Cancer Genome Atlas (TCGA) (Kandoth et al., 2013, Cancer Genome Atlas Network, 2012a), have evaluated these second-order effects, concentrating predominantly on the magnitude of gene expression using microarray technology (Curtis et al., 2012, Perou et al., 2000, Sørlie et al., 2001) or RNA sequencing (RNA-seq) (Shah et al., 2009, Cancer Genome Atlas Research Network, 2012b). They have revealed large-scale disturbances of transcriptional regulation in most cancers, with expression profiles for many hundreds of genes differing from profiles of normal cellular counterparts. Within a tumor type, similarities in transcriptional profiles across individuals allow the disease to be sub-classified into several groups, many of which have biological, therapeutic, and prognostic significance. In some cases, these changes can be correlated with underlying driver mutations, such as *ERBB2* amplification in breast cancer (Sørlie et al., 2001) or specific fusion genes in acute myeloid leukemia (Valk et al., 2004). While these studies have concentrated on mRNA profiles, similar observations are beginning to emerge from studies of microRNA transcription (Dvinge et al., 2013), long non-coding RNA levels, and even expression of pseudogenes (Kalyana-Sundaram et al., 2012).

Although it is a necessary criterion for a driver mutation to directly induce modification of transcript structure, it is not sufficient. Many mutations that do not confer selective advantage, so-called passenger mutations, also generate phenotypic consequences, but consequences of no benefit to the cell. Initial studies correlating RNA-seq data with genomic change in cancer have reported some of these direct effects, especially for coding point mutations or canonical fusion transcripts (Shah et al., 2009), but there has been little systematic effort to describe, measure, and quantify first-order transcriptional consequences across all classes of somatic mutation found in well-annotated cancer genomes.

Here, we report a comprehensive analysis of the primary transcriptional alterations induced by somatic mutation in a set of 23 breast cancers. We found that the genomic variants carried by the cancer cells can have subtle or profound effects on the transcriptome, many of which could not easily be predicted from the genome, many of which amalgamate several in *cis* mutations, and many of which are stably expressed at high levels.

## Results

### Whole-Genome Sequencing and RNA-Seq from 23 Breast Cancer Samples

To understand the inter-relationships between somatic mutation and the transcriptome, we matched RNA-seq data to whole-genome-sequencing data in 23 breast cancer samples. Of these, 14 were primary breast cancers and nine were matched breast cancer cell lines. For the genomes, tumor samples were sequenced to ∼40× coverage and matched normal samples to ∼30× coverage, with somatically acquired substitutions, insertions or deletions (indels), genomic rearrangements, and copy-number changes called by a suite of in-house algorithms. The whole-genome sequencing for the 14 primary breast cancer samples has been described previously (Nik-Zainal et al., 2012a, Nik-Zainal et al., 2012b), although improvements in our bioinformatics algorithm allowed us to update the list of genomic rearrangements (Table S1). The high-coverage genome-sequencing data for eight breast cancer cell lines are reported for the first time here (somatic mutations in Table S2); for the other line (HCC2157), we used exome and low-coverage whole-genome data reported previously (Nik-Zainal et al., 2012b, Stephens et al., 2009).

RNA-seq was performed on the 23 breast cancer samples together with eight organoids freshly isolated from uncultured normal breast milk ducts (Choudhury et al., 2013). We developed a suite of algorithms to exhaustively characterize the cancer transcriptome; in so doing, we aimed to wring maximum detail on the structure of cancer transcripts from RNA-seq data. Previous work has examined gene and mutation expression alone or has focused exclusively on one facet of transcript structure (such as fusion genes or alternative splicing) without allowing for the discovery of multiple or complex events or the involvement of the antisense strand. We implemented a seed-and-extend mapping algorithm to find reads that span different regions of the genome, and then we developed a discordant pair analysis algorithm, drawing these results together with a set of methods to arrange the results into biologically meaningful categories (described in detail in the Experimental Procedures and Figure S1).

The primary advantage of our software pipeline, which we call RNA Architect, is the comprehensive detection of transcriptional alterations, including events missed by other methods. These would include compound events present in *cis*, such as fusion transcripts involving alternative splice forms and exon skips with cryptic splice sites; internal exon shuffling (reusage); post-transcriptional modifications, such as early polyadenylation sites; and non-canonical transcript junctions, for example, fusions between the sense and antisense of different genes or those involving lowly expressed transcripts that are not present in reference databases. While there exists a number of methods for aligning RNA-seq and detecting fusions (Asmann et al., 2011, Chen et al., 2012, Kim and Salzberg, 2011, McPherson et al., 2012, Swanson et al., 2013, Torres-García et al., 2014), there have been few efforts to simultaneously characterize the cancer transcriptome for multiple types of alterations.

### Transcription Derived from Cancer Cells and Stromal Cells

Tumors are comprised of a complex admixture of clonal cancer cells and polyclonal stromal cells. In breast cancer, the proportion of cells deriving from the malignant clone is typically 30%–70%, although the remaining cells encompass endothelial cells, supporting connective tissue, inflammatory cells, lymphocytes, and normal breast epithelium. RNA samples extracted from primary breast cancers, therefore, represent an amalgam of gene expression signatures derived from multiple cell lineages, compounding interpretation.

In females, a randomly selected X chromosome is inactivated in each cell of the inner cell mass of the early blastocyst, and this choice is transmitted through every subsequent cell division. Since cancer cells are derived from a single ancestral cell, all have the same X chromosome inactivated (Fialkow et al., 1981), whereas the polyclonal stromal tissue has a broadly equivalent fraction of cells with maternal or paternal X chromosomes inactivated. As a result, genes undergoing X inactivation with heterozygous germline SNPs are monoallelically expressed in the cancer cells and biallelically expressed in stromal cells (Figure S2).

**Figure 1:**
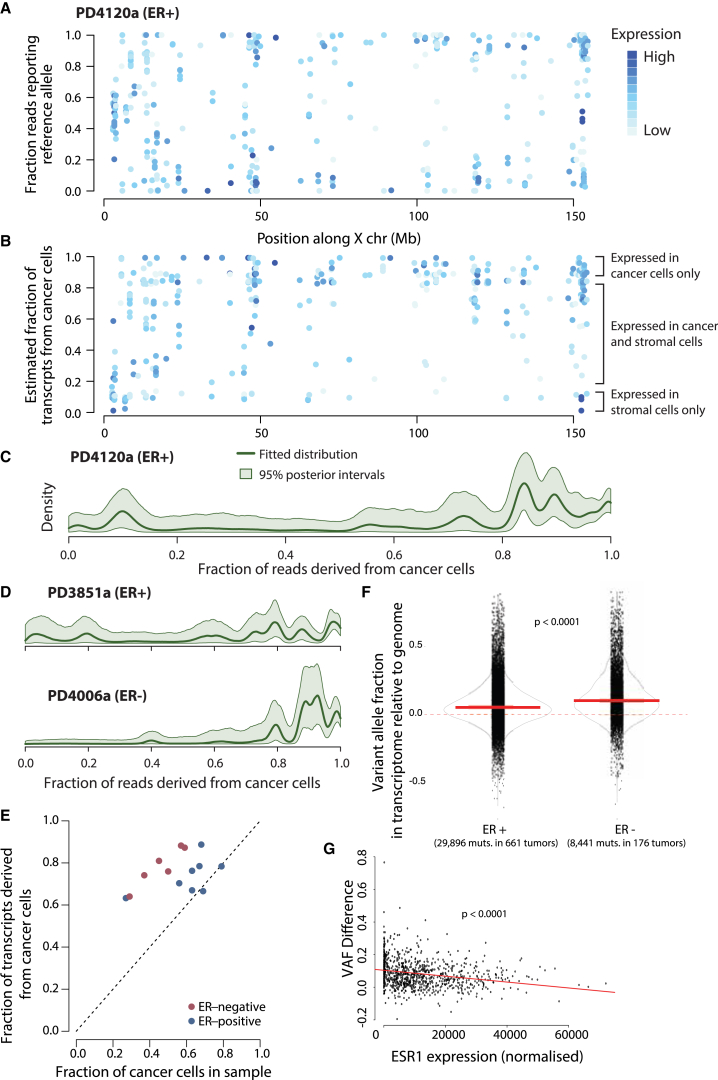
Separating Expression of X-Linked Genes into Stromal and Tumor Compartments (A) Fraction of RNA-seq reads reporting reference allele of heterozygous germline SNPs on the X chromosome in one of the patients (PD4120a). The depth of color reflects the level of expression. (B) Fraction of transcripts derived from tumor cells for each heterozygous germline SNP shown in (A), estimated with a Bayesian Dirichlet process, is shown. (C) Estimated distribution and 95% posterior intervals for relative gene expression in cancer versus stromal cells for PD4120a. The y axis reports the estimated density of genes; the x axis reports the fraction of transcripts for each gene deriving from cancer cells. Thus, the transcripts for most genes in PD4120a are 80%–100% derived from cancer cells and 0%–20% from stromal cells, with only a small peak of genes predominantly expressed from stromal cells. (D) Distributions for several selected primary cancers are shown, as for (C). (E) Overall fraction of transcripts derived from cancer cells (y axis) compared to the estimated proportion on tumor cells in the sample (x axis, estimated from genomic DNA using copy-number profiles) is shown. (F) Increased expression of the mutated allele in ER− as compared to ER+ breast cancer transcriptomes (plotted relative to the genome). Primary breast cancers sequenced as part of TCGA are shown. Plotted on the y axis is the variant allele fraction in the transcriptome, relative to the genome (VAF_diff_). (G) Inverse relationship between each tumors’ expression of Estrogen Receptor 1 (ESR1) and the overall expression of its point mutations (shown as VAF_diff_; −0.2433, p < 0.0001). Using linear regression analysis to model this relationship, we determined that, for every 1% drop in ESR1, ∼15 additional point mutations are expressed.

We identified heterozygous germline SNPs in expressed regions of the X chromosome from the genomic sequencing data across the 14 primary breast cancers, excluding regions that were not diploid in the cancer. From the RNA-seq data, we extracted the number of reads expressing each allele. In PD4120a, for example, 385 heterozygous SNPs on the X chromosome were expressed. The observed reference/variant ratio in the RNA-seq data at each position ranged from transcripts whose expression was exclusively monoallelic through transcripts with skewed ratios to genes that had an approximately equal expression of both alleles (Figure 1A). Respectively, these three scenarios represent genes expressed exclusively in cancer cells, genes expressed in both cancer and stromal cells, and genes expressed exclusively in stromal cells.

We developed a statistical algorithm based on a Bayesian hierarchical Dirichlet process to model the fraction of transcripts along the X chromosome derived from cancer cells (Experimental Procedures). For each heterozygous SNP, the algorithm estimates what fraction of reads covering that base derived from cancer cells, allowing for the uncertainty of whether the reference or variant allele is inactivated in the tumor (Figure 1B; Figure S2B). When amalgamated across SNPs from the whole X chromosome, we can estimate the relative contribution of stromal and cancer cells to transcription as a general distribution (Figure 1C).

We found a considerable portion of transcripts that were exclusively expressed in cancer cells among the 14 patients in which primary breast cancer samples were sequenced (Figure 1D; Figure S2C). Strikingly, many tumors had a set of transcripts that were 80%–90% derived from cancer cells and 10%–20% from stromal cells, whereas there were only small numbers of genes expressed predominantly from stromal cells. We also could integrate all the data for a given patient to estimate the overall fraction of transcripts derived from tumor cells, and we could compare this to the overall fraction of cancer cells in the sample estimated from the genomic DNA (Figure 1E). This indicates that cancer cells contribute a higher fraction of transcripts in the RNA sample than expected for their cellular proportion, indicating that they are more transcriptionally active than the stromal cells. Thus, even though cancer cells comprise, on average, 30%–70% of all cells in a breast tumor, they contribute 70%–90% of all RNA molecules.

Strikingly, it appeared that the magnitude of the difference between transcriptional output of cancer cells and stromal cells was greater in estrogen receptor-negative (ER-ve) tumors than in ER-positive tumors (Figure 1E). The difference in ER-ve also was seen in an independent set of primary breast cancers (661 ER positive and 176 ER-ve), using a larger set of variants (38,337 somatic substitutions; Figure 1F; cosmic census genes with high variant allele fraction difference in ER-ve cancers; Table S3). Further, it appears that the number of mutations expressed in a breast tumor, a measure of its transcriptional output, is significantly associated with the amount of estrogen receptor it expresses (−0.2433, p < 0.0001). That is, tumors with high levels of ER express fewer mutations than cancers with low ER. We formally modeled this relationship and determined that, for every 1% decrease in ESR1 expression, 15 more mutations are expressed in breast cancer (Figure 1G).

### Effects of Point Mutations on Structure of the Transcriptome

We identified all somatically acquired base substitutions in the 23 breast cancers that were in expressed regions, and we compared the fraction of sequencing reads reporting the mutant allele in the transcriptome to that expected from the genome (Figures S3A and S3B). As anticipated, there was a strong overall correlation between the genomic and transcriptomic variant allele fraction (r^2^ = 0.59; p < 0.0001). Overall, 6,980 substitutions were found in exons, of which 4,751 were expressed to a sufficient degree that five or more sequencing reads covered the base. Of the 6,980 variants identified in exonic regions of the 23 samples, 4,152 (59%) had discernible expression in the corresponding transcriptome.

**Figure 2:**
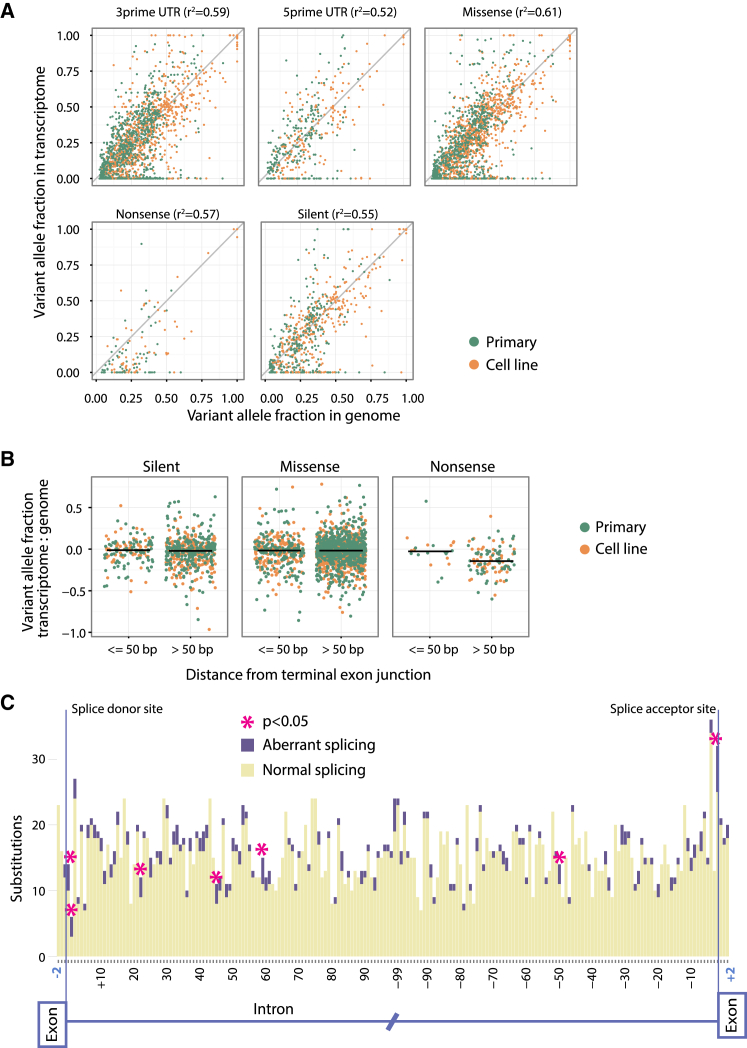
The Effect of Somatic Point Mutations on Expression and Aberrant Splicing (A) Comparison of the variant allele fractions in the transcriptome to the genome, for all classes of point mutation. The squared correlation coefficient between the genome and transcriptome is in parentheses. Only expressed coding changes are shown (five or more times coverage). (B) Variant allele fractions in the transcriptome relative to the genome. Nonsense mutations >50 bp from the terminal 3′ exon-intron junction are the only variants to show a significant difference. (C) Positional effect of mutations on aberrant splicing is shown.

There were some differences in the transcription levels of base substitutions according to the predicted consequence on the protein (Figure 2A). We found that silent, missense, and UTR mutations have the same strong correlation between variant allele fractions in the genome and transcriptome, whereas nonsense mutations have a weaker relationship. Indeed, nonsense mutations had a significantly lower expression than predicted from the genome compared to other classes of mutation (p < 0.0001).

Several reasons could explain the lower expression of nonsense mutations. Nonsense-mediated decay could selectively target transcripts with nonsense mutations for degradation. Nonsense-mediated decay depends on the cell distinguishing a premature termination codon from a proper termination codon. Generally, stop signals in the last exon are considered proper, whereas those appearing >50–55 bp upstream of the last exon-exon junction, and therefore upstream of the exon-junction complex, are more likely to be targeted for nonsense-mediated decay (Nagy and Maquat, 1998). We did find evidence for nonsense-mediated decay, since the decreased allele fraction in transcriptome relative to genome was significantly more pronounced for nonsense mutations if they were >50 bp upstream of the last exon-exon splice junction (p = 0.003; Figure 2B).

Another possible explanation for the low expression of nonsense mutations is that they are tolerated only in genes not expressed in the cancer cells; those occurring in important genes would be subject to negative selection. To explore this possibility, we compared the expression levels from the organoids of normal breast epithelium for genes mutated in the cancer samples. We found no clear-cut differences across the mutation categories for whether the mutated genes were expressed in normal breast epithelial cells (Figure S3C), suggesting that this reason does not explain the lower expression levels of nonsense mutations. Therefore, it appears as if only nonsense-mediated decay explains the lower expression of these mutations.

Point mutations can directly affect RNA splicing, leading to retention of introns (especially for splice donor site mutations), exon skipping (splice acceptor site variants), or enhancement of alternative splice sites (other exonic or intronic variants). We assessed the frequency of alternative splicing events related to somatic base substitutions, where the splice isoform was not present in the normal breast organoids (Figure 2C). We found no excess of abnormal splice isoforms associated with mutations in exons near splice sites. We found that mutations affecting the essential splice sites at +1, +2, and −1 into the intron were the most strongly associated with altered splicing in the given sample (p = 0.002, p = 0.0001, and p = 0.0005, respectively, compared to intronic mutations >100 bp from the nearest exon). Nonetheless, despite this enrichment, the actual fraction of such mutations at essential splice sites that generated detectable abnormal splice isoforms was <25%, suggesting that most such variants do not affect splicing or the transcripts that result are rapidly degraded. Further into the introns, there were some positions at which mutations caused significantly more splicing abnormalities than expected (−49, p = 0.04; +23, p = 0.02; +46, p = 0.01; and +60, p = 0.003). Strikingly, several of these isolated positions coincided with sites of reduced germline polymorphism. For example, the regions from +21 to +26 and from +45 to +50 both showed strongly significant reductions in genetic variation in the germline (Lomelin et al., 2010), suggesting that functional motifs regulating splicing may reside in these sites.

### Direct Effects of Genomic Rearrangements on Transcriptome Structure

Genomic rearrangements contribute to cancer development through several mechanisms, including changing the copy number of a gene or genes, altering the regulatory apparatus of a gene, and reorganizing the exon sequence within a gene or between two genes. To evaluate effects of genomic rearrangements on transcriptome structure, we classified somatically acquired structural variants across two variables: type of rearrangement (deletion, tandem duplication, inverted, or interchromosomal) and whether genes were involved at either side of the breakpoint (gene-to-gene, same or opposite orientation; gene-to-intergenic; within gene, same or different introns; or local genomic complexity, where more than one rearrangement affected one or other gene). For each rearrangement, we identified any aberrant transcript arising from the genes involved, excluding any splice form seen in the normal breast organoids or the Ensembl database.

**Figure 3:**
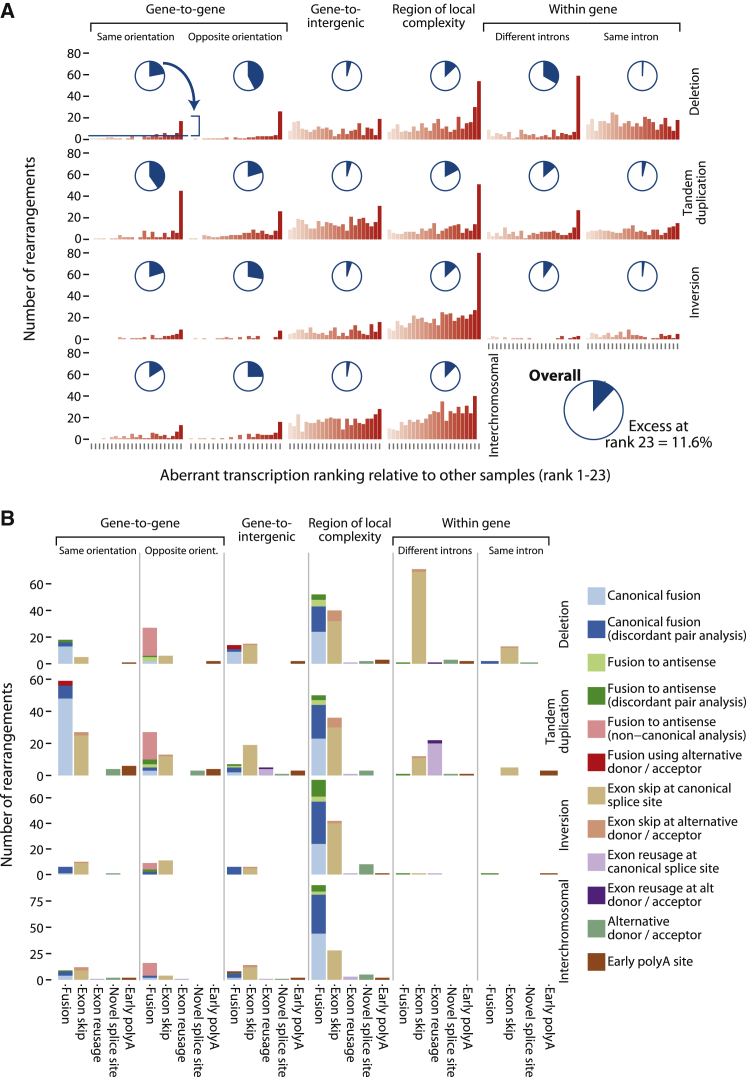
The Transcriptional Consequences of Structural Rearrangement All rearrangement types and their position with respect to genes are shown as a matrix in both panels. Transcriptional disruptions caused by each rearrangement type are shown within the matrix. (A) Number of rearrangements causing aberrant transcription. Normalized aberrant transcription levels were contrasted between the sample that contained the rearrangement and all others. Plotted is the aberrant transcription ranking of the rearranged sample relative to all others for the same genes (red bars). The pie charts show the fraction of all rearrangements of that type that are excess in the final rank compared to the number expected under a uniform distribution. (B) Types of aberrant transcriptional events caused by rearrangements are shown.

Even in cancer samples without rearrangements affecting a given gene, we often found evidence for previously undocumented transcripts, such as novel splice forms, read-through transcripts, and non-canonical splice acceptor or donor sites. It is, therefore, difficult to argue categorically for a given rearrangement that an abnormal transcript arises as a direct consequence of the genomic change. Instead, since we are more interested in the overall patterns of abnormal transcription caused by somatic mutation, we studied the excess of aberrant transcription associated with the different categories of genomic rearrangement. The normalized expression level of aberrant transcripts was ranked for the sample in which the rearrangement was found, relative to aberrant transcripts in the other 22 cancer samples (Figure 3A). If a given rearrangement had no effect on transcription, then the ranking would be effectively uniformly distributed across ranks 1–23, whereas those rearrangements that caused significant alterations to transcriptome structure would garner the highest rank.

There is a clear excess of genomic rearrangements with the maximum ranking for aberrant transcription. Using maximum likelihood methods, we estimated that this excess represents 11.6% (95% confidence interval, 10.4%–12.8%) of 4,234 genic rearrangements identified in these samples (Supplemental Experimental Procedures); that is, 11.6% of somatically acquired genomic rearrangements affecting genes are associated with evidence of aberrant transcription beyond the background rate in breast cancer. This varied by the pattern of genes at the breakpoint, with particularly high rates observed for intragenic rearrangements leading to alterations of exon order but minimal evidence for aberrant transcription arising from rearrangements confined to a single intron within a gene.

We observed a number of different patterns of aberrant transcription (Figure 3B). These included fusion transcripts between two genes, alternative splicing events, exon reusage, and premature polyadenylation. To some extent, the alterations in transcript structure could be predicted by the underlying genomic rearrangements, such as exon skips caused by intragenic deletions, but in many cases the abnormalities were rather surprising. In the following sections, we review the transcriptional consequences associated with each of the major classes of genomic rearrangement.

### Within-Gene Rearrangements

Across the 23 breast cancer samples studied, we identified 631 intrachromosomal rearrangements confined to a single intron of a gene, mostly deletions and tandem duplications (358 and 192, respectively). Of these, we believe that very few had discernible consequences on transcriptome structure, since there was no apparent excess of such rearrangements generating the highest rank for transcriptional aberration across samples (Figure 3A). For the 38 rearrangements with highest rank, the most common effect on transcript structure was to skip an exon (69%; Figure 3B).

As expected, rearrangements that went across different introns of the same gene had a considerably greater effect on transcript structure than those confined to one intron (Figure 3A). In general, the transcriptome reflected the rearranged gene structure in an entirely predictable way, with deletions causing exon skips and tandem duplications causing exon reusage (Figure 3B). Of 341 genomic rearrangements involving different introns of the same gene, 84 had the highest rank for transcriptional abnormality. Of these 84, 23 (27%) caused multiple disruptions in the transcriptome at the same gene, mostly alternative splice isoforms. Particularly common were exon skips of not just deleted exons but neighboring exons as well. We found a complex transcriptional abnormality in the histone H3-lysine-4 (H3K4)-methyltransferase *MLL3* (*KMT2C*) involving an exon reusage using a cryptic donor (Figure S6A). Follow-up experiments using TCGA data revealed an additional 13 samples with abnormalities in *MLL3* (Figure S6B).

### Gene-to-Gene Rearrangements in the Same Transcriptional Orientation

**Figure 4:**
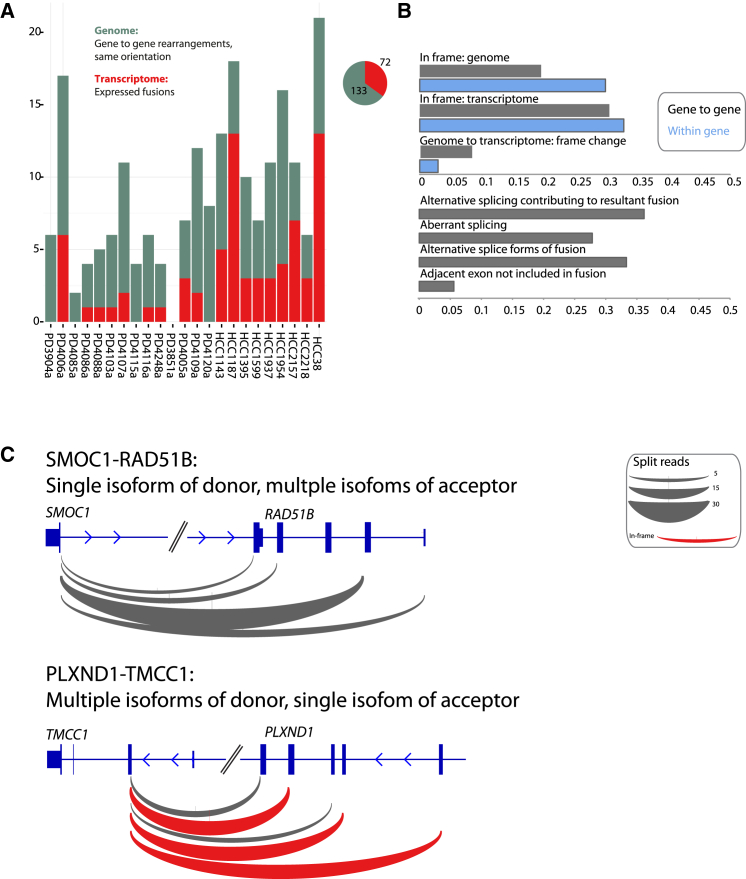
Rearrangements between and within Genes (A) Fusions caused by rearranged genes in the same orientation are shown. (B) Proportion of rearrangements predicted to lead to an in-frame event contrasted to the proportion actually expressing in-frame transcripts (top). Characteristics of expressed fusions (bottom) are shown. (C) Many fusions are expressed in multiple isoforms.

We identified 205 somatic rearrangements that juxtaposed one or more exons of two protein-coding genes in the same transcriptional orientation; these would be predicted to generate fusion genes. Overall, 70 (34%) of these were expressed (Figure 4A). As seen with the within-gene rearrangements, the transcriptome structure was generally as predicted from the genomic rearrangement, although more than one splice isoform was present in 20 of the 70 rearrangements, increasing the range of transcripts observed (Figure 4B). The only recurrent fusion transcript that we observed in this cohort was between *NCOA7* and *TRMT11*, adjacent genes on chromosome 6, caused by tandem duplications in HCC1954 and PD4005a (Figure S4).

We examined the protein-reading frame of transcripts arising from gene fusions and within-gene rearrangements that spanned more than one intron (Figure 4B). In 133 of 501 (27%) such events, the resulting exon structure would be predicted to generate an in-frame gene from transcript isoforms reported in Ensembl; we found RNA-seq reads supporting these in-frame transcripts in 35 of 133 (26%). Of the 368 rearrangements predicted to be out of frame or involve the non-coding UTR, we found evidence for in-frame transcripts in 25 (7%). In many cases, the in-frame transcript was more heavily expressed than the canonical, out-of-frame transcript, suggesting that nonsense-mediated decay may be acting on the latter (Figure 4C). Overall then, these data indicate that 60 of 501 (12%) of genomic rearrangements reordering exons of one or two genes in the same orientation have the potential to generate transcripts encoding in-frame proteins. Many of these are expressed at appreciable levels, mostly driven by the upstream regulatory apparatus.

### Gene-to-Gene Fusions in Opposite Transcriptional Orientation

**Figure 5:**
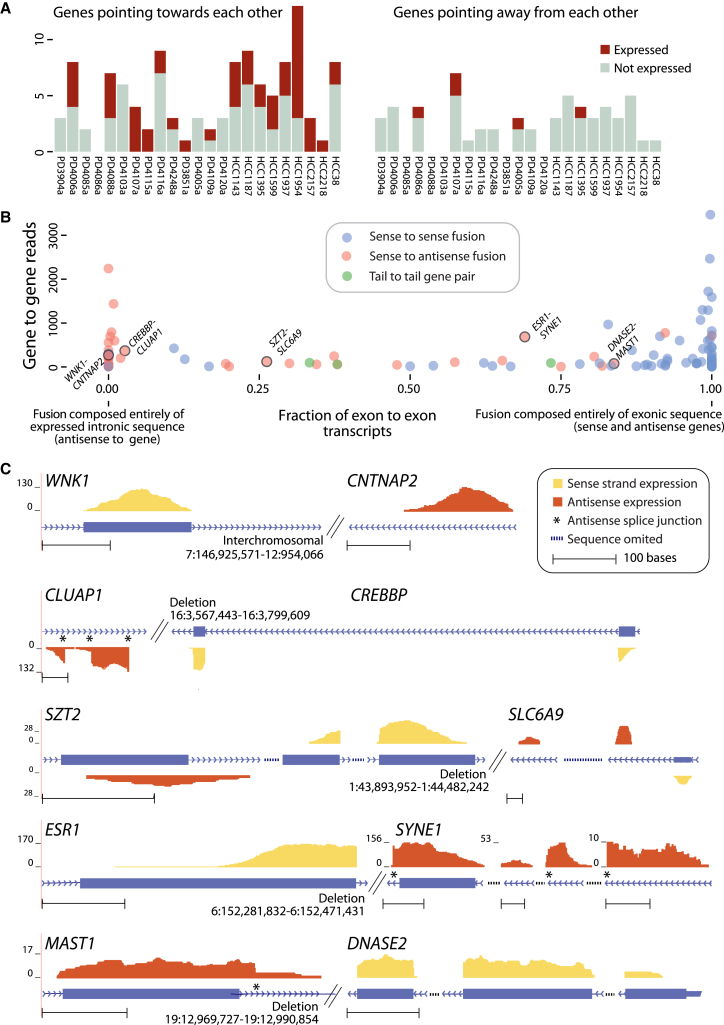
Antisense Expression Caused by Rearranged Genes in Opposite Orientation (A) Stacked bar plot shows the number of expressed transcripts per sample resulting from gene fusions in opposite orientation. (B) The diversity of chimeric transcripts produced by gene-to-gene rearrangements. The expression level of each transcript is plotted on the y axis. Tail-to-tail gene pairs (green) are rarely expressed, whereas, surprisingly, sense-to-sense and sense-to-antisense fusions show similar levels of expression (blue and red, respectively). Transcripts are placed on the x axis according to the type of read joining the two genes. Genes adjoined by exonic reads are plotted to the right on the x axis, and genes brought together only by exon-to-intron reads are on the left. (C) Examples of productive, stable antisense fusion transcripts. Plotted on the y axis are the read depths supporting the fusion. Hatched lines indicate rearrangement breakpoints. In most cases, we observed a single donor gene, which expresses sequence from its sense strand (yellow), and a single acceptor gene, which expresses sequence from its antisense strand (red). Rarely are both promoters used, leading to reciprocal sense-antisense fusions (both genes express sense and antisense sequence). The fusions *SZT2*-*SLC6A9* and *SLC6A9*-*SZT2* are examples of a reciprocal pair. In general, antisense transcripts display features of traditional exons: they are stably expressed, around 200 bp, and are frequently spliced at GT-AG splice sites (asterisks).

We would expect half of the genomic rearrangements linking two genes to join them in opposing orientation, which would be split equally between gene pairs pointing inwardly at each other and gene pairs pointing away from each other. In the former, the 5′ regulatory apparatus and transcriptional start site of both genes would be retained, and they could start transcripts that would extend into the partner gene on the antisense strand. We identified 171 somatic rearrangements generating gene-to-gene fusions in opposite orientation, of which 114 were pointing inward (5′-to-5′ orientation). While there was not much evidence of aberrant transcription arising from 3′-to-3′ fusions, we found an unexpectedly high frequency of stable transcription at gene pairs pointing inwardly (Figure 5A).

In total, 50 (44%) of all 5′-to-5′ rearrangements generated transcripts that fused the sense portion of one gene with novel exons on the antisense strand of the partner gene. Mostly, the novel transcribed sequence from the antisense strand of the distal gene mapped to intronic regions, although a few fusions did generate transcripts that partially or fully overlapped with exons (Figure 5B). This is to be expected since splice sites are directional, so the GT…AG structure of an intron is not recapitulated on the antisense strand. However, where one might have expected the reads derived from the antisense strand to be rather scattered, the antisense exons were, in fact, surprisingly fixed (Figure 5C). That is, the antisense component of the fusion transcript tended to reuse the same latent splice acceptor and donor sites on the antisense strand. These were almost always associated with consensus GT-AG splice signals. For a small number of examples, multiple antisense exons were recurrently included in the transcript. None of these novel antisense exons was seen in the absence of the given 5′-to-5′ rearrangement, suggesting that it is the genomic rearrangement that unmasks the latent transcriptional potential of these regions.

It is unclear what functional potential these sense-to-antisense gene fusions might have. Notably, we found an example involving the estrogen receptor, *ESR1*, which generated transcripts linking the sixth exon into a multiply spliced antisense transcript of *SYNE1* (Figure 5C). Fusion transcripts involving the same intron of *ESR1* are recurrent in breast cancer, and there is evidence they have important functional consequences, largely conferred by the C-terminally truncated estrogen receptor (Li et al., 2013). There is also a rearrangement that fuses the first 20 exons of the transcriptional co-activator *CREBBP* to the antisense strand of *CLUAP1*. *CREBBP* is a well-known cancer gene that can be targeted by inactivating point mutations (Pasqualucci et al., 2011) or, in leukemias, involved in canonical fusion genes (Camós et al., 2006).

### Gene-to-Intergenic Rearrangements

**Figure 6:**
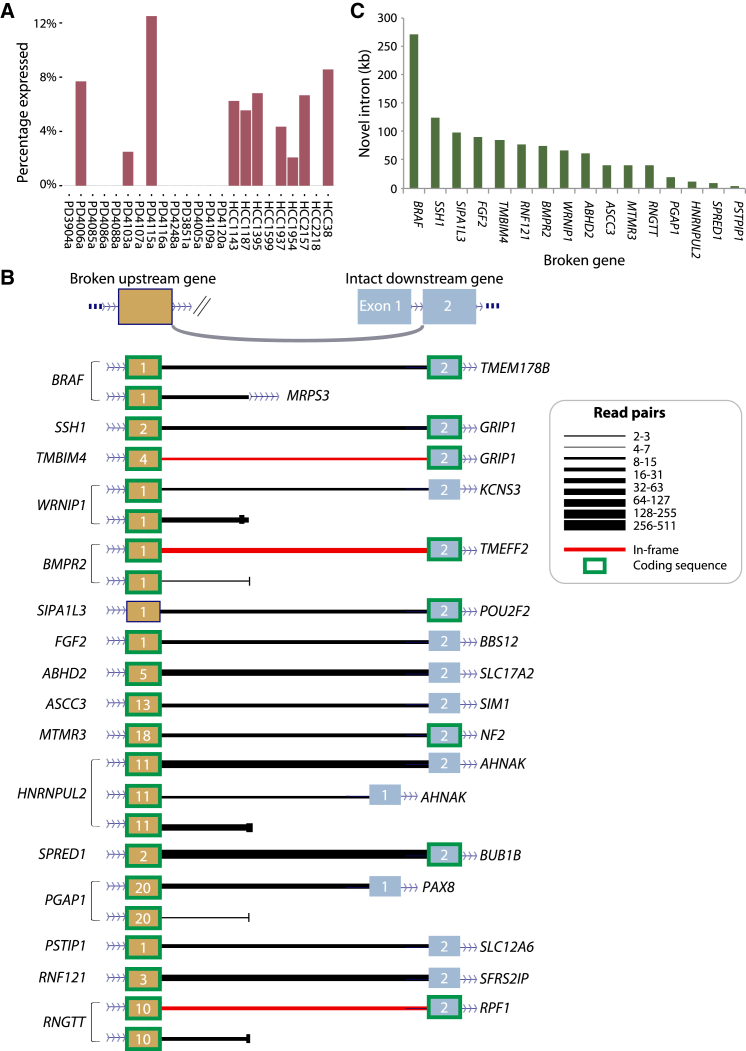
Non-canonical Fusions Caused by Gene-to-Intergenic Breakpoints (A) Percentage of gene-to-intergenic rearrangements causing fusions is shown. (B) Length of the intron created is shown. (C) Genes involved in non-canonical fusions. We observed 18 fusions where a broken gene (donor) splices to another gene (acceptor) that is itself unbroken and often distant. These fusions can be highly expressed (width of line) and cause in-frame transcripts (red line).

We identified 473 genomic rearrangements that joined the 5′ portion of a gene to an intergenic region and 461 rearrangements linking 3′ ends of genes to intergenic space (Figure 6A). As seen with the 3′-to-3′ gene-to-gene fusions, in the absence of promoters, only one 3′ gene-to-intergenic rearrangement led to a detectable RNA transcript. In contrast, 16 (3.4%) of 5′ gene-to-intergenic rearrangements led to stable expression of abnormal transcripts related to the rearrangement.

The predominant transcripts that resulted from these 5′ gene-to-intergenic rearrangements were fusions linking the 5′ portion of the broken gene to exon 2 of the first intact, sense gene downstream of the breakpoint (Figure 6B). Occasionally, splicing into novel intergenic exons or into exon 1 of the downstream gene was observed, but, compared to splicing into exon 2, these transcripts were infrequent and represented minor RNA species. In general, the first exon of a gene commences with the transcription start site and, therefore, does not contain a splice acceptor site, explaining why 5′ gene-to-intergenic rearrangements fuse into exon 2. Since the first exon of many genes carries the ATG that initiates translation, many of these gene-to-intergenic rearrangements could translate into bona fide fusion proteins. Indeed, we identified three fusion transcripts caused by gene-to-intergenic rearrangements that were potentially in frame (Figure 6B). The length of the novel intron created by these transcribed gene-to-intergenic fusions was typically in the 50- to 100-kb range, but it could be as high as 250 kb (Figure 6C).

### Regions of Local Complexity

**Figure 7:**
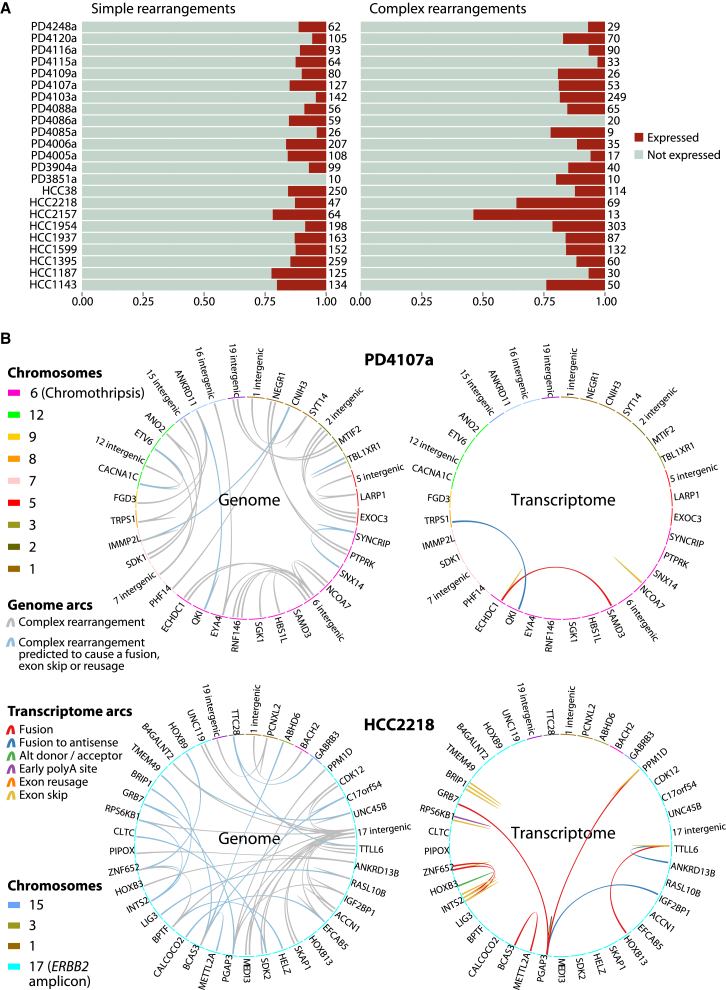
Regions of Local Complexity Give Rise to Unique Transcriptional Consequences A region of local complexity is any gene footprint that contains two or more genomic rearrangements. Local complexity can occur in regions of chromothripsis and high-level amplification. (A) Proportion of simple and complex rearrangements that lead to an expressed transcript, grouped by sample, is shown. (B) Regions of local complexity and their transcriptional consequences. Two samples’ regions of complexity are shown as pairs of Circos plots. The genomic events one would predict to be expressed are highlighted (blue arcs). Often the tumors do not express these events, or they amalgamate multiple *cis* rearrangements and express a transcript that combines genes only indirectly linked to another.

We defined a region of local complexity as any gene footprint that contained two or more genomic rearrangements. Typically, these represented sites of extensive genomic amplification, such as around *ERBB2* or *CCND1*, or they were regions of chromothripsis, a mutational process generating tens to hundreds of localized genomic rearrangements in a one-off catastrophic event (Stephens et al., 2011). Given the complexity of the genomic changes in many of these regions, a surprising number of rearrangements led to measurable transcriptional consequences (Figure 7A). Indeed, when compared with genes hit by simple rearrangements, the fractions of rearrangements from regions of local genomic complexity giving aberrant transcripts were broadly similar. This suggests that the regulatory apparatus enabling transcription initiation remains at least partially intact in many of these heavily rearranged regions and that the genomic structure supports the production of stable transcripts.

We found that the transcripts that arose in these regions often represented an integration across multiple rearrangements (Figure 7B; Figure S5). In PD4107a, for example, we found a fusion transcript that linked *QKI* to the antisense strand of *TRPS1* (blue arc, Figure 7B), which was, in fact, driven by two in *cis* genomic rearrangements linking *QKI* to *ANKRD11* and then *ANKRD11* to *TRPS1*. Due to the massive number of rearrangements sometimes found in these regions of local complexity, there can be a considerable degree of aberrant transcription. In PD4103a, for example, among the hundreds of clustered rearrangements localized to a small number of genomic regions, we found 12 different fusion transcripts as well as seven alternatively spliced isoforms driven by within-gene rearrangements (Figure S5).

## Discussion

The disturbed transcriptional landscape of cancer cells results from three main forces: (1) direct, primary consequences of somatic mutation; (2) coordinated, secondary gene expression changes resulting from altered cellular signaling, transcriptional regulation, and chromatin landscape; and (3) general loss of transcriptional fidelity, manifesting as shorter 3′ UTRs (Mayr and Bartel, 2009), retained introns, *trans*-splicing (Li et al., 2008), and so on. Here we have concentrated on dissecting the immediate impact that the repertoire of somatic mutations has on the transcriptome in breast cancer, exploring the rules that govern how the transcriptional machinery interprets somatic mutation. In some ways, this is the most straightforward analysis of a cancer transcriptome to perform; the causation chain is short and, in theory, predictable.

One striking conclusion of the analysis is that transcription, once started, will attempt to complete. We found an unexpectedly high number of transcripts resulting from structural variants that sow the 5′ seeds of a gene, namely upstream enhancers, promoter, and first few exons, into seemingly infertile ground, such as intergenic space or the antisense strand of another gene. Indeed, in our data, the fraction of such events generating productive transcription was not dissimilar to that observed for rearrangements predicted to cause canonical gene fusions. In the case of gene-to-intergenic rearrangements, the transcriptional machinery can scan many tens of kilobases in search of a splice acceptor site, often contributed by the second exon of a downstream intact gene in the same orientation. For sense-to-antisense fusions, the sense transcript often splices into novel exons within the gene footprint of the antisense gene. In one example, this generated a truncated version of the estrogen receptor gene *ESR1*. Recently, it has been reported that fusion transcripts arising from breaks in the same intron of *ESR1* are recurrent in breast cancer and can confer resistance to endocrine therapy (Li et al., 2013).

Another important observation is that the transcriptional output of breast cancer cells is greater than the surrounding stromal cells. Using a method that accounts for differences in tumor purity, we found a striking anti-correlation between ER levels and the number of expressed mutations. That is, tumors with high levels of ER express fewer mutations than cancers with low ER. We formally modeled this relationship and determined that, for every 1% decrease in ER expression, 15 more mutations are expressed. As a breast cancer loses estrogen receptor expression and becomes more transcriptionally active, it is more likely to actually express its complement of somatic mutations. For example, we found that *TP53* mutations are more likely to be expressed in ER− than ER+ breast cancers (Figure S7B). While speculative, this is of interest to researchers in the field of immunotherapy, since somatic mutations can act as neoantigens that trigger host immune responses. There are studies reporting strong associations between the number of neoantigens and response to immunotherapy, and our data suggest that such mutations are more likely to be expressed in ER-ve or ER-low tumors.

It is a necessary condition of a somatic mutation to be oncogenic that it induces some transcriptional consequence, but it is far from sufficient. We found that 59% of exonic point mutations are expressed and 11.6% of genomic rearrangements (balanced and unbalanced) hitting a gene footprint generate aberrant transcripts. These aberrant transcripts are polyadenylated, stable, and have the potential to generate protein products. In the case of cancer, even those that generate proteins will be mostly inconsequential to cell biology, although there will be some that are oncogenic. In the case of species evolution, however, such a high proportion of genomic rearrangements generating stable fusion transcripts, novel exons, and splicing isoforms could readily provide a substrate for further genomic evolution over many generations.

### Statistics

#### Statistical Model for Analyzing Allele-Specific Expression of Heterozygous Germline SNPs on the X Chromosome

For every heterozygous SNP on the X chromosome, we have an observed count of transcripts expressing the reference allele and a count for those expressing the variant allele. For each SNP, we do not know whether the reference allele is on the active X chromosome (Xa) or the inactive X chromosome (Xi) in the tumor cells. We further assume that, in the contaminating stromal cells, the expected proportion of cells with the reference allele on Xa is 50%. We model these data using a hierarchical Bayesian model, where the distribution of the fraction of reads deriving from tumor cells from across all genes follows a Dirichlet process.

We define N as the number of heterozygous germline SNPs on the X chromosome in a given sample and $n_{i},\;i=1,\ldots ,N$ as the total number of reads across SNP i, of which $y_{i}$ report the reference allele. Then $y_{i}∼Bin\left(n_{i},p_{i}\right)$, where $p_{i}$ is the expected proportion of reads reporting the reference allele. Here $p_{i}$ follows a mixture model depending on whether the reference allele is on Xa or Xi.

$$f\left(p_{i}\right)=\lambda_{i}\pi_{i}+\left(1-\pi_{i}\right)/2,$$

where $\pi_{i}$ is the fraction of transcripts derived from tumor cells for SNP i, and

$$\lambda_{i}=\{\begin{array}{c} 1,\;\text{if}\;\text{reference}\;\text{allele}\;\text{is}\;\text{on}\;\text{Xa}\\ 0,\;\text{if}\;\text{reference}\;\text{allele}\;\text{is}\;\text{on}\;\text{Xi}\text{.} \end{array}$$

We let $\lambda_{i}∼Bern\left(0.5\right)$ as the prior, with $\pi_{i}∼DP\left(\alpha P_{0}\right)$. We use the stick-breaking representation of the Dirichlet process as follows:

$$P=\sum_{h=1}^{\infty }\omega_{h}\delta_{\pi_{h}},\;\text{with}\;\pi_{h}∼P_{0},$$

where $\delta_{\pi }$ is a point mass at π, and $\omega_{h}$ is the weight of the *h*th gene expression cluster (that is, effectively the proportion of genes for which the fraction of transcripts deriving from tumor cells is π. To capture the stick-breaking formulation, we let $\kappa_{h}=V_{h}\prod_{l<h}\left(1-V_{l}\right)$, with $V_{h}∼Beta\left(1,\alpha \right)$. We set a practical maximum number of clusters, C, as 40. As priors, we set $P_{0}∼U\left(0,1\right)$ and α∼Γ(0.01,0.01).

To model the posterior distribution of the Dirichlet process, we use Gibbs sampling as described below.

##### Step 1: Allocating Each Gene to One of the Clusters

We set indicator variables, $S_{i}\epsilon \left\{1,2,\ldots ,C\right\}$, to denote allocation of gene i to a cluster. The posterior distribution of these variables is therefore

$$\mathrm{Pr}\left(S_{i}=h\text{|}-\right)=\frac{\left(V_{h}\sum_{l<h}\left(1-V_{l}\right)\right)\left(\left(\begin{array}{c} n_{i}\\ y_{i} \end{array}\right)p_{h,i}^{y_{i}}{\left(1-p_{h,i}\right)}^{n_{i}-y_{i}}\right)}{\sum_{r=1}^{C}\left(V_{r}\sum_{l<r}\left(1-V_{l}\right)\right)\left(\left(\begin{array}{c} n_{i}\\ y_{i} \end{array}\right)p_{r,i}^{y_{i}}{\left(1-p_{r,i}\right)}^{n_{i}-y_{i}}\right)},$$

where h=1,2,…,C and $p_{h,i}=\lambda_{i}\pi_{h}+\left(1-\pi_{h}\right)/2$.

##### Step 2: Updating the Stick-Breaking Weights

These are conditionally conjugate beta posterior distributions as follows:

$$\left(V_{h}\text{|}-\right)∼Beta\left(1+\sum_{i=1}^{N}1\left(S_{i}=h\right),\alpha +\sum_{i=1}^{N}1\left(S_{i}>h\right)\right),$$

where h=1,…,C−1 and $V_{C}=1$.

##### Step 3: Updating the Fraction of Transcripts Deriving from Tumor Cells

We want to generate draws from the posterior distribution of $\left(\pi_{h}|-\right)$. We use a Metropolis-Hastings algorithm with beta proposal distribution to do this. So,

$$\begin{array}{c} \mathrm{Pr}\left(\pi_{h}\text{|}-\right)\alpha \left[\begin{array}{c} {\left(\left(1+\pi_{h}\right)/2\right)}^{\sum_{i:S_{i}=h}\lambda_{i}y_{i}}\;x\;{\left(\left(1-\pi_{h}\right)/2\right)}^{\sum_{i:S_{i}=h}\left(1-\lambda_{i}\right)y_{i}}\;x\\ {\left(\left(1+\pi_{h}\right)/2\right)}^{\sum_{i:S_{i}=h}\left(1-\lambda_{i}\right)\left(n_{i}-y_{i}\right)}\;x\;{\left(\left(1-\pi_{h}\right)/2\right)}^{\sum_{i:S_{i}=h}\lambda_{i}\left(n_{i}-y_{i}\right)} \end{array}\right]\\ ={\left(\left(1+\pi_{h}\right)/2\right)}^{\sum_{i:S_{i}=h}\left(2\lambda_{i}y_{i}-\lambda_{i}n_{i}+n_{i}-y_{i}\right)}{\left(\left(1-\pi_{h}\right)/2\right)}^{\sum_{i:S_{i}=h}\left(y_{i}-2\lambda_{i}y_{i}+\lambda_{i}n_{i}\right)} \end{array}$$

and the proposal distribution is $g\left(x|x^{\prime }\right)=Beta\left(x^{\prime }d,\;d\left(1-x^{\prime }\right)\right),$ where d is a scale parameter to be fine-tuned by trial and error to achieve a reasonable acceptance/rejection proportion.

Then the importance ratio for the jump from $\pi^{\left(t-1\right)}$ to the proposed $\pi^{*}$ is given by

$$\frac{\text{Pr}\left(\pi^{*}|-\right)}{\text{Pr}\left(\pi^{\left(t-1\right)}\right)}\frac{g\left(\pi^{\left(t-1\right)}|\pi^{*}\right)}{g\left(\pi^{*}|\pi^{\left(t-1\right)}\right)}.$$

##### Step 4: Updating the Indicator Variables of Whether the Reference Allele Is on X_a_ or X_i_

With a little algebra, this can be shown to follow

$$\mathrm{Pr}\left(\lambda_{i}=1\text{|}-\right)=1/\left(1+{\left(\frac{1-\pi_{h}}{1+\pi_{h}}\right)}^{2y_{i}-n_{i}}\right).$$

##### Step 5: Updating the Hyperparameter

The posterior distribution for α is

$$\left(\alpha \text{|}-\right)∼\text{Γ}\left(C+A-1,\;B-\sum_{l=1}^{C-1}\text{log}\left(1-V_{l}\right)\right),$$

where the prior is α∼Γ(A,B).

#### Statistical Analysis of Relationship between Variant Allele Fractions in the Genome and Transcriptome for Different Classes of Somatic Substitution

We fitted linear mixed-effects models to the variant allele fractions. The variant allele fraction of reads reporting the mutant allele was the dependent variable. The random effect was the patient from whom the RNA sample derived, allowing for inter-individual differences in both the intercept and slope of the line correlating genomic with transcriptomic variant allele fraction. The fixed effects were the genomic variant allele fraction and class of variant, with the main hypothesis of interest being whether the relationship between genomic and transcriptomic allele fraction differed across the classes of variant. This was tested by adding class of variant-by-slope interaction terms to the model, using likelihood ratio tests to assess for improved fit. Models were fitted using maximum likelihood methods.

## Experimental Procedures

### Detection of Changes to Gene Transcript Structure

We developed a suite of tools for the analysis of cancer RNA-seq data, called RNA Architect, comprised of several algorithms.

RNA Architect’s central component is a seed-and-extend algorithm used to find reads that span disparate regions of the genome. These are junction reads that provide the breakpoint between non-adjacent loci of fusion genes or alternative splice forms. We describe below how we obtained these highly informative split reads by pre-filtering; grouping the reads into common events; and then classifying, annotating, and filtering the high-confidence events (Figures S1A and S1B).

(1) Pre-filter. We first removed any read that mapped normally to a genic region in the human genome (build 37). That is, the read was fully contained in an exon or was split across adjacent exons. We also excluded read pairs if one end mapped to the mitochondria or if it contained two or more unknown nucleotides (N). The pre-filtering step was performed by aligning all reads to a transcriptome reference containing known exons derived from Ensembl (using a modified version of BWA 0.5.9).
(2) Index. Having removed all of the reads involving known transcriptional events, we ran a sensitive alignment of the remaining reads. We first scanned the human genome, and we built an index containing the positions of all exons of all protein-coding genes, pseudogenes (including polymorphic pseudogenes), and processed transcripts. We used a word size of 9 bp as this provided the best balance between sensitivity and run time.
(3) Shatter reads and align k-mers. We then shattered the 75-bp reads into k-mers (13 bp), yielding five k-mers of equal size and one short k-mer containing the 3′ end of the read (the lowest quality portion of Illumina reads). All k-mers were then aligned to the indexed genes without allowing for any mismatches.
(4) Merge and extend. K-mers that map to adjacent positions were merged into a single fragment. The k-mers that did not map are those that spanned a breakpoint (inter- or intragenic). We then extended mapped fragments into their unmapped neighbors, one base at a time. We iteratively added bases to the k-mers, and we removed them from the unmapped neighbors, until all fragments mapped to a unique location and we successfully resolved the breakpoints. At this point we allowed mismatches (SNPs, substitutions, or indels) only if they were proceeded by a 2-bp perfect match to the reference.
(5) Clean. Most fusions and aberrant splice breakpoints were resolved using the methods outlined above. However, if a fragment remained unmapped, we tried to map it using modified parameters. If there existed homologous sequences between both edges of the breakpoints, leading to ambiguity over where the breakpoint should be positioned, we chose the breakpoint pair that yielded a junction spanning canonical donor-acceptor sequences (GT-AG).
(6) Annotate. Having resolved the breakpoints of all fusion genes, aberrant splice forms, and compound events, Architect writes junction coordinates into a database (SQLite) and the split reads into a binary alignment file (BAM). Each event, which represents the junction of two or more expressed sequences that are not normally found to be adjacent, was ranked across a number of measures, including whether the event is seen in normal samples or in the reference genome; the number of reads supporting the event (total and unique); the sequence context of the junction (i.e., whether canonical donor-acceptor sequences are used); the average number of unique bases per read; and the number of reads mapped in the direct and reverse complemented orientation. We then implemented separate processes that used these measures to arrange the events into the following biologically meaningful categories:
  (A) Exon skips. A junction between two non-adjacent exons from the same transcript, not found in normal organoids (less than two reads) or in Ensembl, where the first exon is 5 prime to the second exon. Exon skips were annotated to the Ensembl transcript that involved the fewest number of exons lost. We required a split forward read and a split reverse read to call an exon skip. We subcategorized exon skips into those that involved the canonical edges of the exons and those that involved a cryptic splice site (either 5′ or 3′ of the donor or acceptor site).
  (B) Exon reusages. A junction between two non-adjacent exons from the same transcript, not found in normal organoids (less than two reads) or in Ensembl, where the first exon is 3 prime to the second exon. Exon reusages were reported only if there were no Ensembl transcripts that would explain the junction between the exons. We recorded the number of exons reused and required a split forward read and a split reverse read to call an exon reusage. We subcategorized exon reusages into those that involved the canonical edges of the exons and those that involved a cryptic splice site (either 5′ or 3′ of the donor or acceptor site).
  (C) Alternative donors and acceptors. A junction between two exons that begins 5′ or 3′ from the canonical exon-intron border. Alternative donor and acceptor sites can involve the extension of the exon into the adjacent intron or the reduction of the exon. The junction must not be found in normal organoids (less than two reads) or in Ensembl, and it must involve canonical donor-acceptor sequences (GT-AG).
  (D) Early polyadenylation sites. A junction between the exon of a protein-coding gene and a non-templated run of adenines (or thymines if mapped to the opposite strand), which does not occur at the canonical edge of the gene’s UTR. The junctions must be >10 bp away from the canonical edge, and they must not be found in normal organoids (less than two reads) or in Ensembl. We subcategorized early polyadenylation sites into those in UTRs and those in coding exons.
  (E) Fusions genes. A junction between the exons of two protein-coding genes that is not found in control samples. We had two approaches to finding fusion gene pairs: (1) using aberrant junctions that were split across a breakpoint joining two genes, and (2) using read pairs that were fully mapped (i.e., not split) to both genes. The first approach used the seed-and-extend algorithm outlined above. The second approach used a bespoke discordant-pair analysis algorithm, which involved remapping the initially unmapped reads as singletons, re-pairing them together, and evaluating whether there existed clusters of read pairs aligning to two genes in a consistent manner.

We only reported high-confidence fusion genes. These had to have been detected by both the seed-and-extend algorithm and the discordant-pair algorithm or detected by the discordant-pair algorithm alone with extremely high ranking. This ranking was determined by evaluating the following: how many reads map to other locations in the transcriptome (multi-mappings), the consistency of the mapping positions (percentage of coefficient of variation <25), the number of unique reads in each gene (more than five), and the consistency of the discordant read pairs’ orientation (<5% inconsistent). We excluded reads where one end maps to the mitochondria. We subcategorized fusion genes based on the relative orientation of the genes involved (same orientation, or in opposite orientation [antisense]) and whether the fusion also involved a cryptic splice site.

## Author Contributions

A.S., M.R.S., and P.J.C. designed the study, analyzed the data, and wrote the manuscript. K.R., F.F., R.A., S.N.-Z., S.D., A.R, Y.S.J., S.L.C., M.R., E.P., H.R.D., P.S.T., P.V.L., D.C.W., and J.M. contributed towards genomic and transcriptomic tool development and analysis. L.M. developed RNA-seq methods. S.M. was project coordinator. E.A., C.H., V.B., S.A.J.R.A., T.S., O.G., A.V.-S,. O.M., S.B., A.F., A.L., Å.B., G.T., A.L.R., A-L.B.-D., and K.P. contributed towards analyses. All authors discussed the results and commented on the manuscript.

## Accession Numbers

The accession numbers for the raw sequence data reported in this paper are The European Genome-phenome Archive under accession numbers EGAD00001002236 and EGAD00001002237 (https://www.ebi.ac.uk/ega/, hosted by the European Bioinformatics Institute [EBI]).

## Supplemental Information

Document S1. Supplemental Experimental Procedures and Figures S1–S7

Table S1. Genomic Rearrangements

Table S2. Somatic Mutations in Breast Cancer Cell Lines

Table S3. COSMIC Cancer Genes Containing Mutations with Positive Variant Allele Fraction Difference in ER-ve Cancers

Document S2. Article plus Supplemental Information
