# Supplementary material for: Direct Transcriptional Consequences of Somatic Mutation in Breast Cancer
Source: Cell Rep. 2016 Aug 4;16(7):2032–46. doi: 10.1016/j.celrep.2016.07.028 (PMC4987284; doi:10.1016/j.celrep.2016.07.028)
Supplement: Document S1. Supplemental Experimental Procedures and Figures S1–S7 [file mmc1.pdf]

Cell Reports, Volume *16*

Supplemental Information

Direct Transcriptional Consequences of Somatic Mutation in Breast Cancer

Adam Shlien, Keiran Raine, Fabio Fuligni, Roland Arnold, Serena Nik-Zainal, Serge Dronov, Lira Mamanova, Andrej Rosic, Young Seok Ju, Susanna L. Cooke, Manasa Ramakrishna, Elli Papaemmanuil, Helen R. Davies, Patrick S. Tarpey, Peter Van Loo, David C. Wedge, David R. Jones, Sancha Martin, John Marshall, Elizabeth Anderson, Claire Hardy, ICGC Breast Cancer Working Group, Oslo Breast Cancer Research Consortium,, Violetta Barbashina, Samuel A.J.R. Aparicio, Torill Sauer, Øystein Garred, Anne Vincent-Salomon, Odette Mariani, Sandrine Boyault, Aquila Fatima, Anita Langerød, Åke Borg, Gilles Thomas, Andrea L. Richardson, Anne-Lise Børresen-Dale, Kornelia Polyak, Michael R. Stratton, and Peter J. Campbell

Supplementary Methods

Analysis of variant allele fraction differences between the transcriptome and genome in TCGA data

To validate our finding, we calculated differences in transcriptional output in TCGA's breast cancer cohort. Aligned BAM files for 980 breast cancer samples with both RNA-Seq and exome sequencing were downloaded from CGHUB (https://cghub.ucsc.edu/) using GeneTorrent. PCR duplicates for both exome and transcriptome were removed using SAMtools. The position of somatic mutations, in MAF file format, and gene expression values (using the RSEM method) were obtained from https://tcga-data.nci.nih.gov/. Additional clinical covariates were obtained from cBioPortal (http://www.cbioportal.org/). All putative mutations were re-annotated using Annovar (release 2013Aug23) and all potential germline variants were removed (present in NCBI dbSNP Human build 142). Finally, 70,071 exonic/splicing substitutions present in the 980 RNA-Seq and WES paired samples were considered for further analysis. Mutations in the 5' or 3' UTRs were excluded. Mutated loci were considered not expressed, and therefore excluded from this analysis, if the total coverage was less than five reads, or the number of reads supporting the mutated base was less than five reads.

These substitution mutations were evaluated in a number of ways, including by measuring the proportion of reads reporting the mutation in the transcriptome (variant allele fraction or VAF) and subtracting it from the same measure in the genome (i.e. $VAF_{difference} = VAF_{transcriptome} - VAF_{genome}$). We used linear regression to model the relationship between the amount of ESR1 expressed by a tumor and the VAFdiff of its mutations.

We classified TCGA breast cancers into known subtypes (Luminal B, Luminal A, HER2-related and triple negative) by immunohistochemistry as per Blows et al (PLOS Med 2010).

Estimating the excess of rearrangements with maximum rank for aberrant transcription

We use a maximum likelihood approach to estimate the excess of rearrangements at highest rank. Basically, we allow the ranks to be distributed as a multinomial process with probabilities of rank~{π,π,⋯,π,π+τ}, where we are interested in estimating τ. It is straightforward to show that the maximum likelihood estimator for τ is given by:

$$\hat{\tau} = \max\left\{0, \left((C-1)r_C - \sum_1^{C-1} r_i\right) \Big/ (C-1)\sum r_i\right\}$$

where C is the number of samples (different ranks) and r_i is the number of rearrangements garnering the ith rank. Bootstrapping of the observed counts across all possible ranks was used to estimate the 95% confidence intervals for the point estimates.

Detection of genomic rearrangements

Paired-end maps were generated using a new in-house algorithm that will be published separately (J. Marshall et al., manuscript in preparation). Briefly, discordantly mapped read pairs were filtered against BWA read pileup loci, repeat features and mitochondrial sequences in GRCh37. Additionally alternative mapping locations were evaluated to assess whether both reads could be aligned to an alternative location as a concordant pair. Remaining discordant read pairs were clustered to generate a putative list of rearrangements with respect to the GRCh37 reference genome. Candidate rearrangements found in paired normal blood DNA analyses, or previously confirmed by PCR to be germ line in other studies, were removed. These steps produced a paired-end map cured from the majority of the artefacts resulting from BWA-mapping and from putative germ line variants.

In this manuscript we only report high confidence rearrangements for which we have successfully resolved the breakpoint. To find the breakpoints we first determined the window surrounding rearrangements using the average and maximum insert size of each BAM file. We then looked for reads where one end mapped within this window and the other end was unmapped. Unmapped reads were realigned to the genome (BLAT, using optimised parameters). Realigned reads that accurately mapped within the both windows of a rearrangement were grouped together, and finally each putative breakpoint was evaluated by measuring the distance between the breakpoint region and the breakpoint, and the coefficient of variation of the breakpoint position themselves (ideally, there is no variability at the position).

Validation of changes to gene transcript structure

We validated our RNA-Seq results by using replicate RNAs, comparing the junctions to existing datasets, using RNA pull-down sequencing, and by manual inspection.

Sample HCC1599 was run as a technical replicate. A new library was created, sequenced and analysed using the same algorithms. One hundred percent of the genomic rearrangements causing an exon skip with the highest rank were found to lead to the same event in the replicate transcriptome (5/5). Of the three genomic rearrangements involving two genes in the same orientation, which were previously found to cause an expressed fusion, two caused the same event in the replicate transcriptome and one was missed in the replicate. We compared the in-frame fusions to those we previously reported (Stephens et al. Table 3). Of the 14 in-frame fusions found in both analyses, which had previously been validated by RT-PCR, we identified 10 expressed fusions in the new RNA-Seq data as well as many other others not reported in the previous data set.

Supplementary Figures Legends

Supplementary Figure 1. RNA Architect, a suite of algorithms for the analysis of cancer RNA-Sequencing. Related to Figure 3.

(A) Overview of RNA Architect's seed-and-extend and discordant pair algorithm.

(B) Statistics from a representative sample that has been run through this pipeline.

(C) All samples sequenced at high depth, and there is no association between coverage and percentage of expressed mutations.

(D) Similar levels of expressed mutation found in TCGA data.

Supplementary Figure 2. Estimating the proportion of reads derived from the tumour and the stromal cells. Related to Figure 1.

(A) Comparison of variants from the active and inactive X chromosome.

(B) Observed fraction of reads reporting reference allele vs. the posterior probability of the reference allele deriving from the active X chromosome. The depth of colour reflects the level of expression.

(C) Estimated distribution and 95% posterior intervals for relative gene expression in cancer versus stromal cells for ER+ and ER- breast cancers.

Supplementary Figure 3. Related to Figure 1; Figure 2.

(A) Increased expression of the mutated allele in ER- as compared to ER+ breast cancer transcriptomes (plotted relative to the genome).

(B) Variant allele fraction in genome compared to the transcriptome, for all samples including cell lines.

(C) Absence of negative selection in nonsense mutations. Comparison of expression levels from the organoids of normal breast epithelium for genes mutated in the cancer samples.

Supplementary Figure 4. A recurrent in-frame fusion between *TRMT11* and *NCOA7* in two breast cancers. Related to Figure 3; Figure 4.

A tandem duplication on chromosome 6 joins the 5' end of *TRMT11* with the 3' end of *NCOA7.* In both samples the fusion is in-frame and highly expressed as shown by the numerous junction reads (split reads) between *TRMT11* exon 11 and *NCOA7* exon 13 in sample PD4005a, and *TRMT11* exon 6 and *NCOA7* exon 7 in sample HCC1954.

Supplementary Figure 5. Regions of local complexity in breast cancer sample PD4103a. Related to Figure 7. One sample's regions of complexity are shown as pairs of Circos plots, for the genome and transcriptome. The genomic events one would predict to be expressed are highlighted (blue arcs). The tumour does not express all of these events, or multiple *cis* rearrangements have been amalgamated and expressed as a single transcript that combines genes only indirectly linked to another.

Supplementary Figure 6. Compound event in the gene *MLL3*. Related to Figure 3.

(A) A tandem duplication in the genome within the footprint of *MLL3,* an established breast cancer gene, results in a complex aberrant transcript involving the reusage of exons and the activation of an alternative donor site. The reads from TopHat support junctions between the canonical exon edges (red arcs) only

whereas RNA Architect identifies the compound event (horizontal lines represent split reads).

(B) Aberrant *MLL3* transcripts. Shown are novel isoforms of *MLL3* found in TCGA breast cancers (n=980). Data were reanalysed and reprocessed using our pipeline. We compared each putative aberrant junction in *MLL3* to 1,277 normals from 30 tissue types and excluded anything found in these samples (GTEX).

Supplementary Figure 7. Transcriptional output of ER-positive and ER-negative breast cancers. Related to Figure 1.

(A) The expression of mutations differs within known molecular subgroups of breast cancer. Samples were grouped using available clinical data (Supplementary Methods), into known molecular subgroups. Plotted on the Y-axis is the VAF_{diff}. The pie charts, shown each subgroup, depicts the percentage of mutations expressed.

(B) Differences in expression of TP53 missense mutations between ER+ and ER- breast cancers.

(C) Expression of common mutated genes in ER-negative and ER-positive cancers.

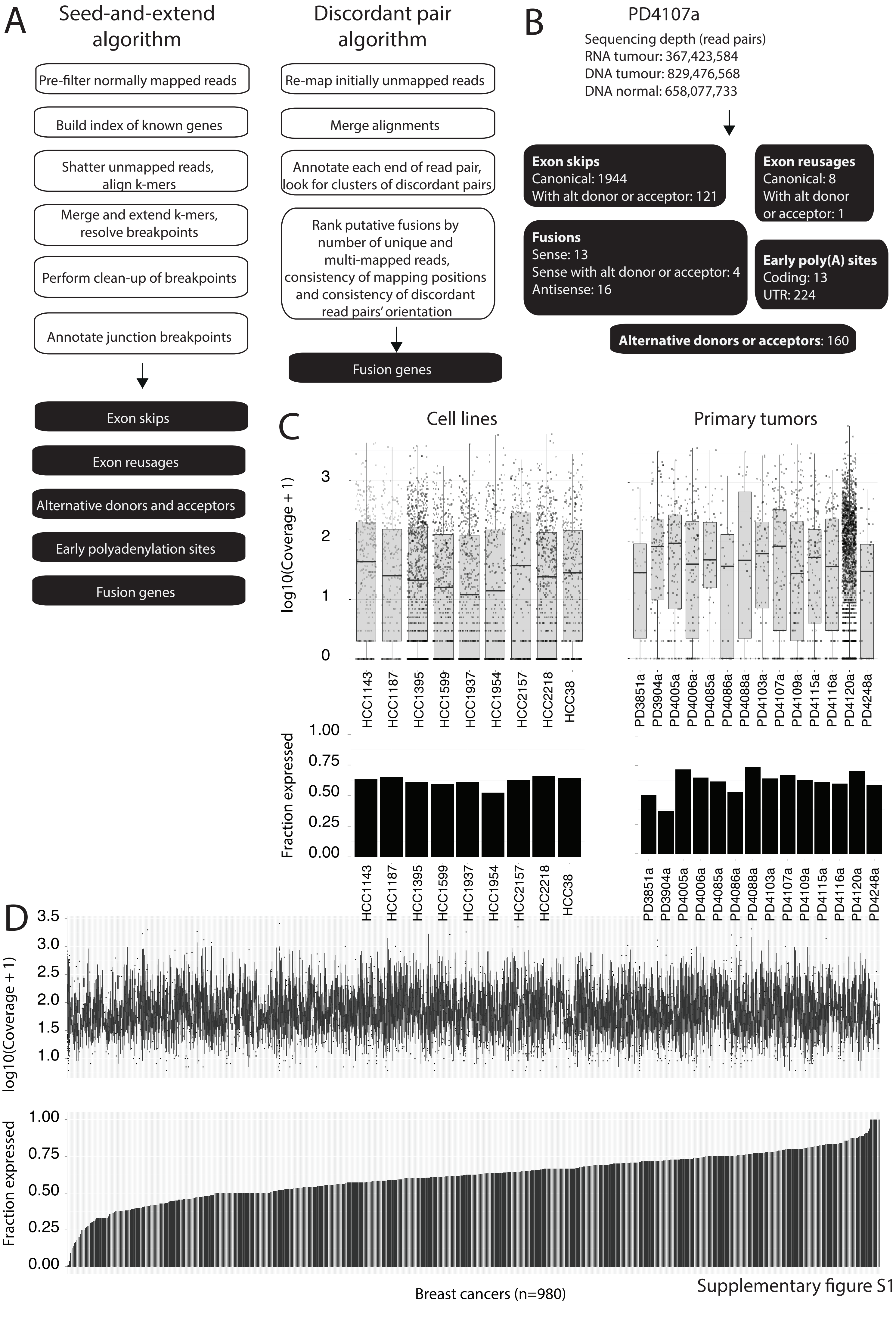

Supplementary figure S1

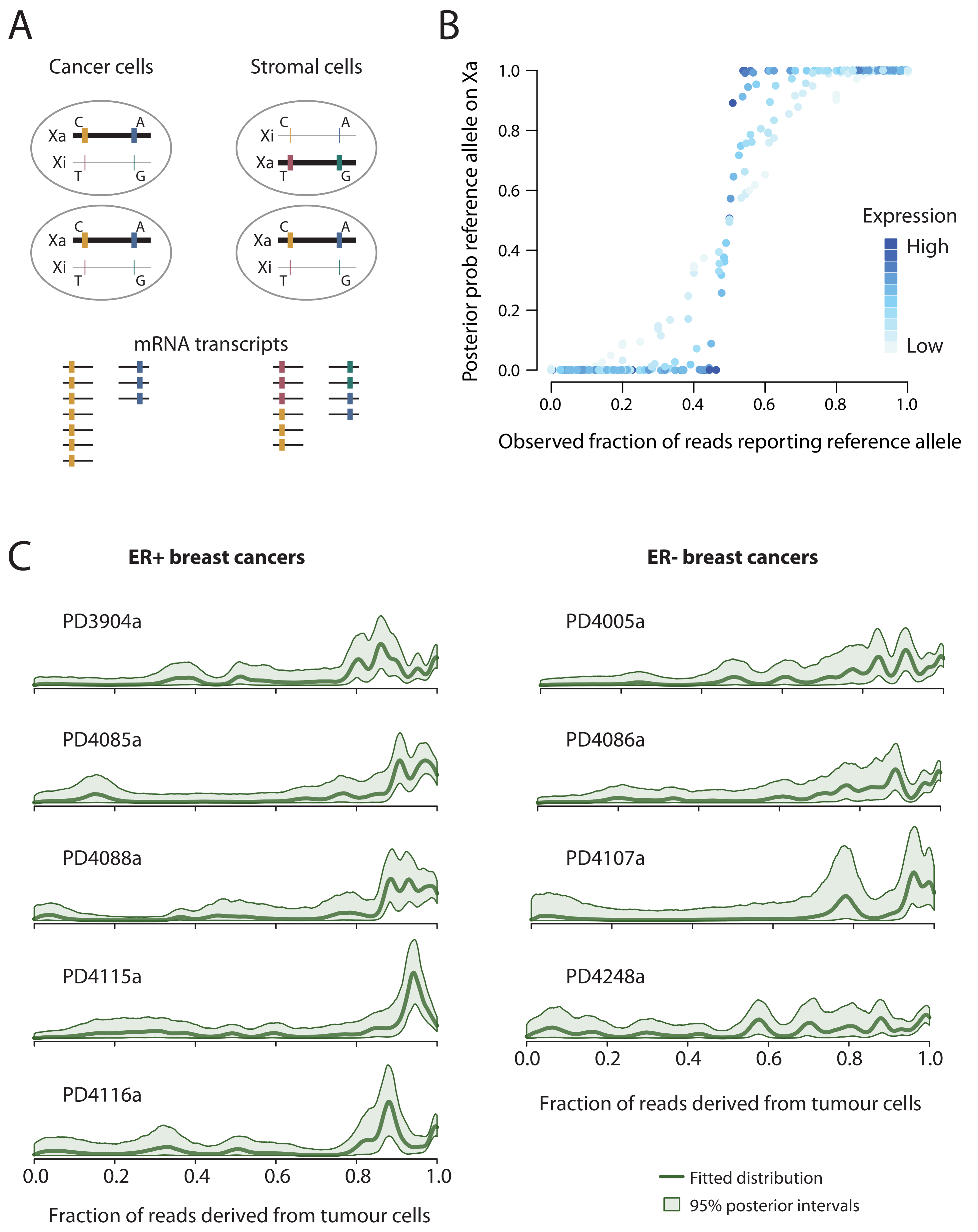

Supplementary figure S2

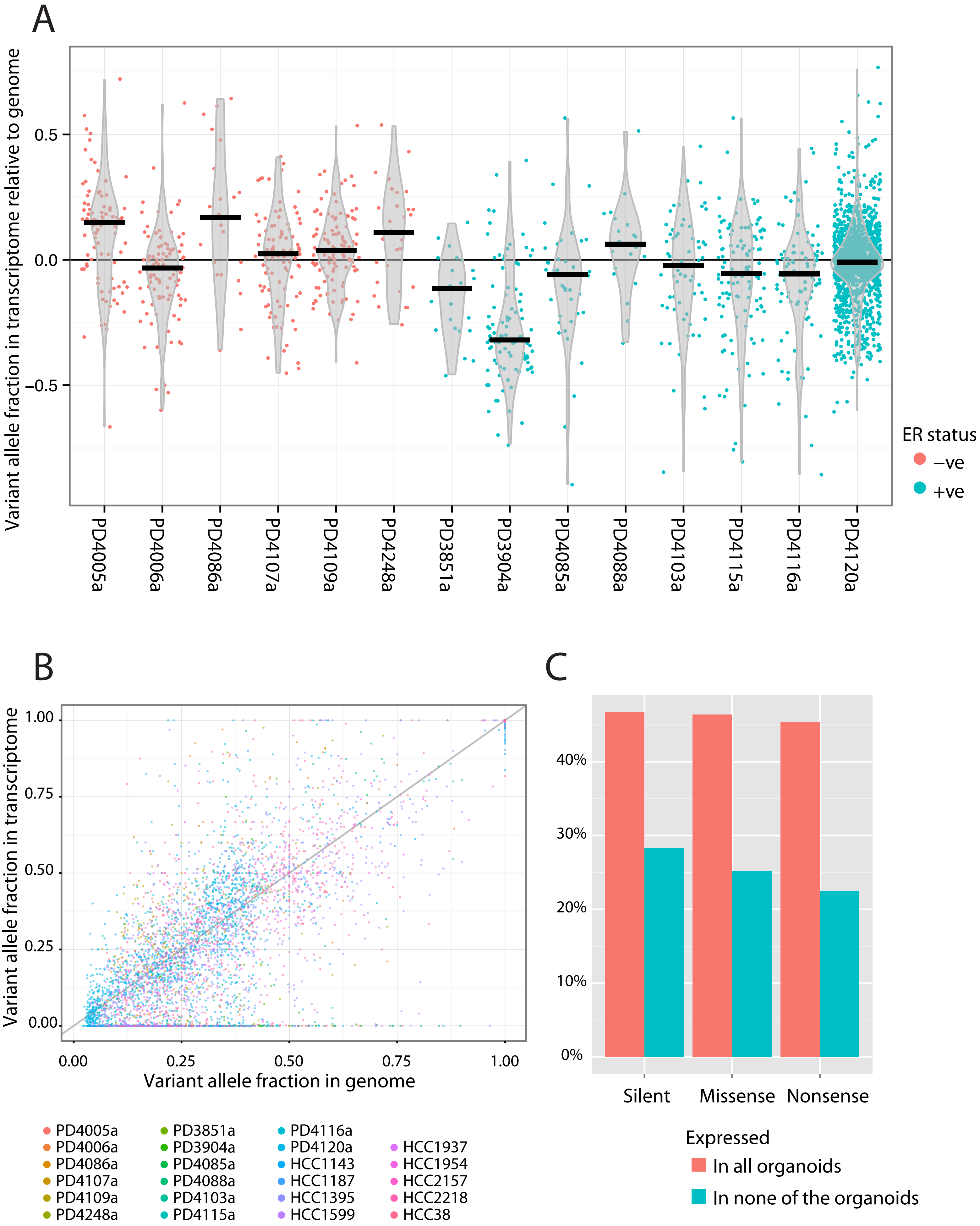

Supplementary figure S3

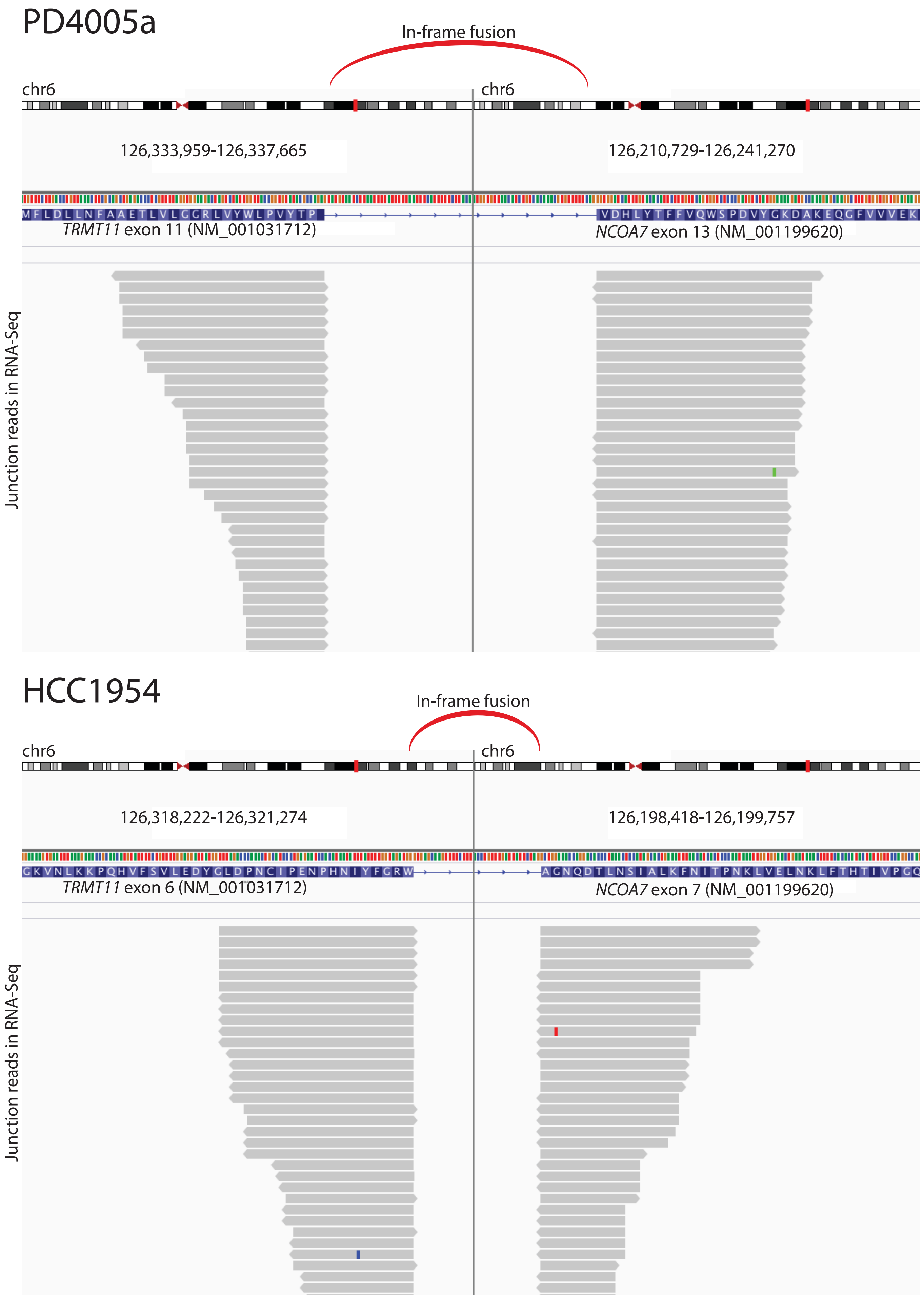

Supplementary figure S4

PD4103a

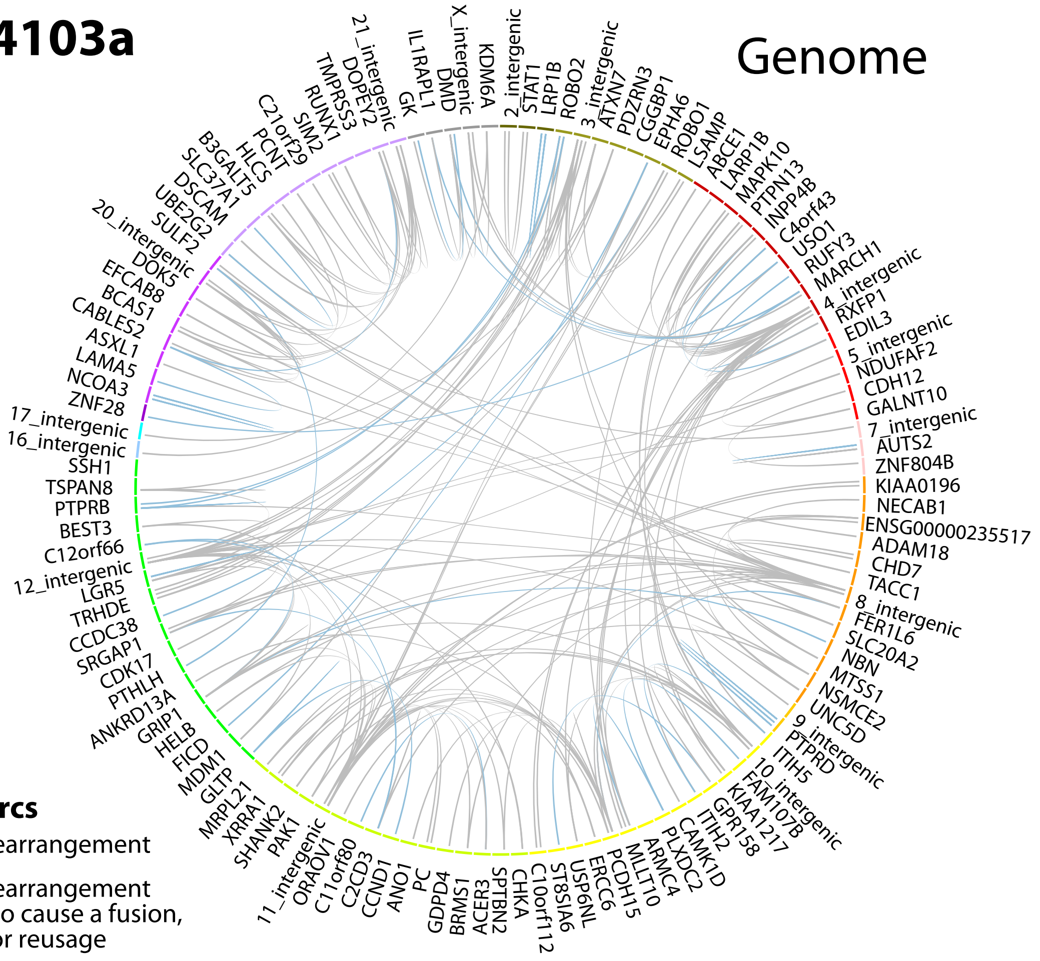

Genome arcs

- Complex rearrangement
- Complex rearrangement predicted to cause a fusion, exon skip or reusage

Transcriptome arcs

- Fusion
- Fusion to antisense
- Alt donor / acceptor
- Early polyA site
- Exon reusage
- Exon skip

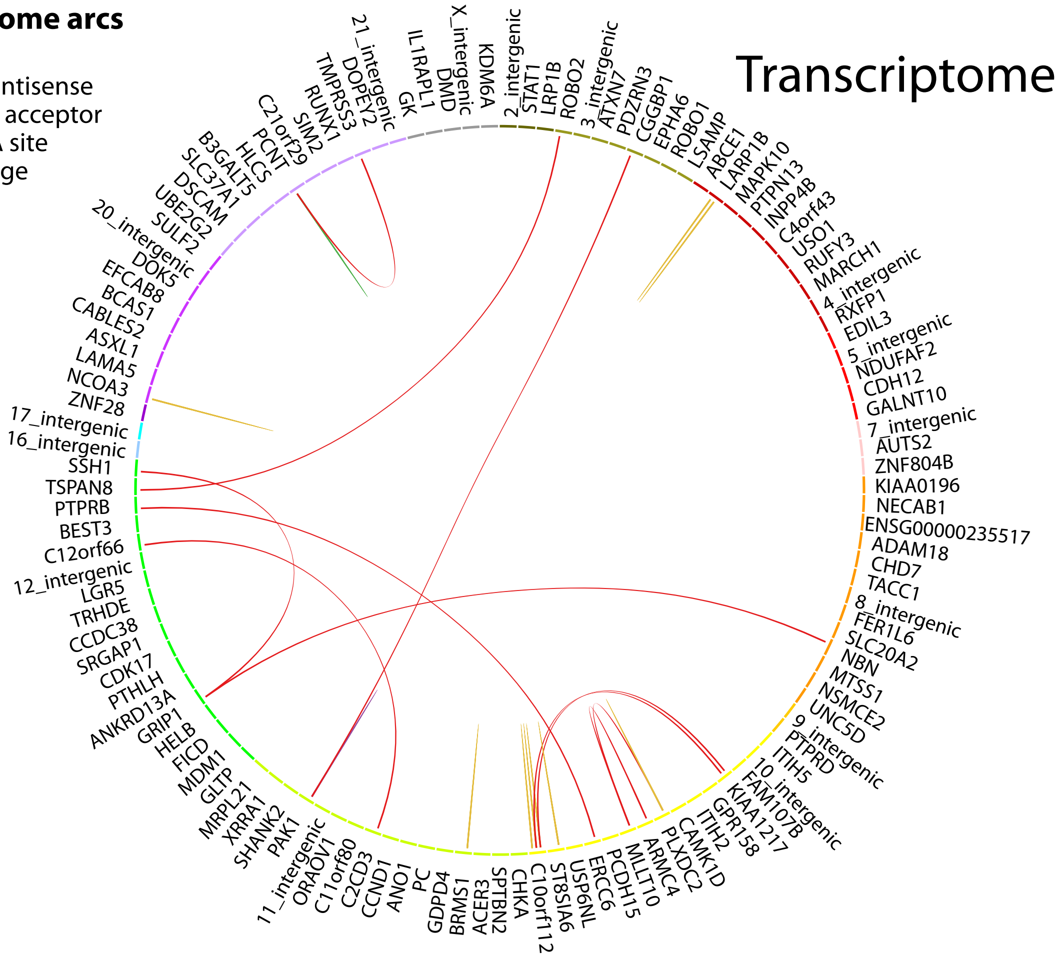

Supplementary figure S5

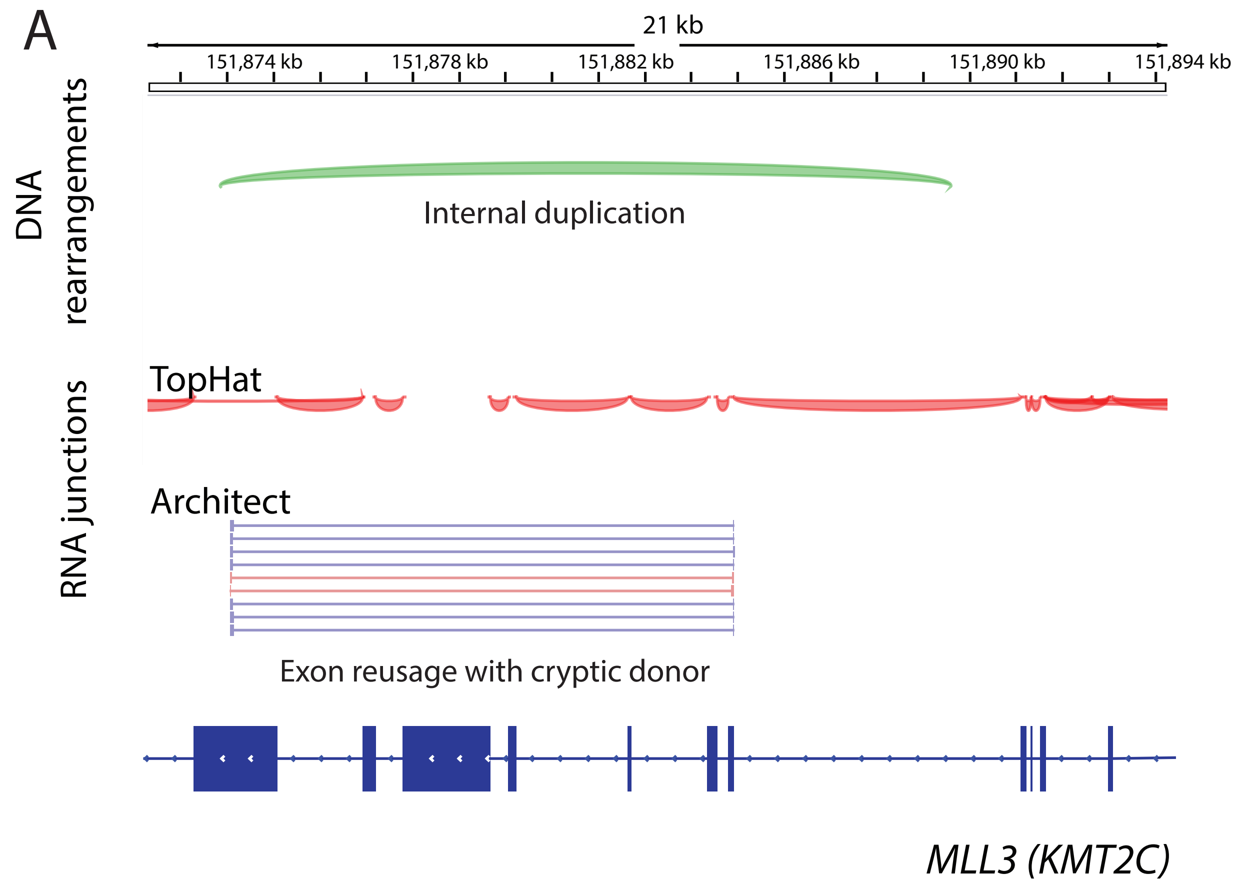
A
21 kb
151,874 kb
151,878 kb
151,882 kb
151,886 kb
151,890 kb
151,894 kb
DNA rearrangements
Internal duplication
RNA junctions
TopHat
Architect
Exon reusage with cryptic donor
MLL3 (KMT2C)

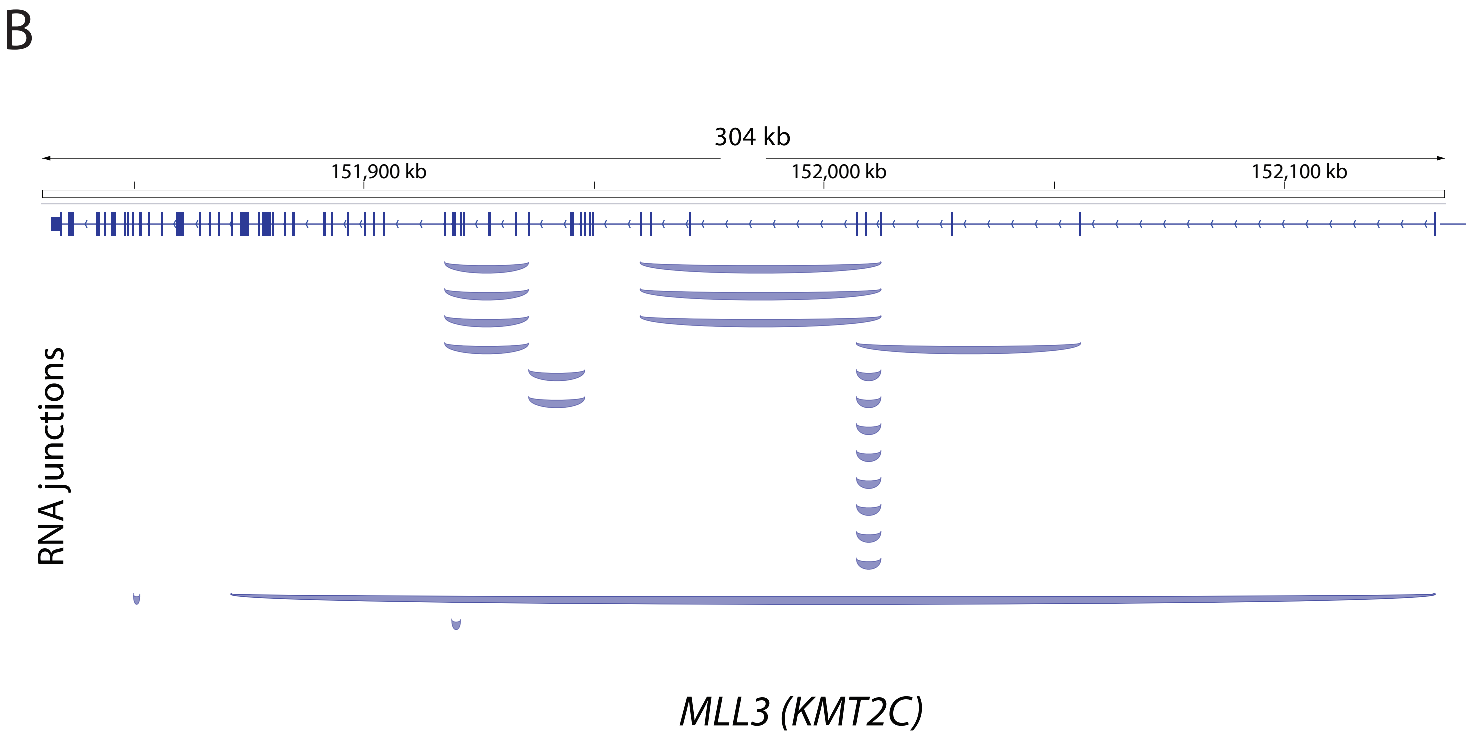
B
304 kb
151,900 kb
152,000 kb
152,100 kb
RNA junctions
MLL3 (KMT2C)

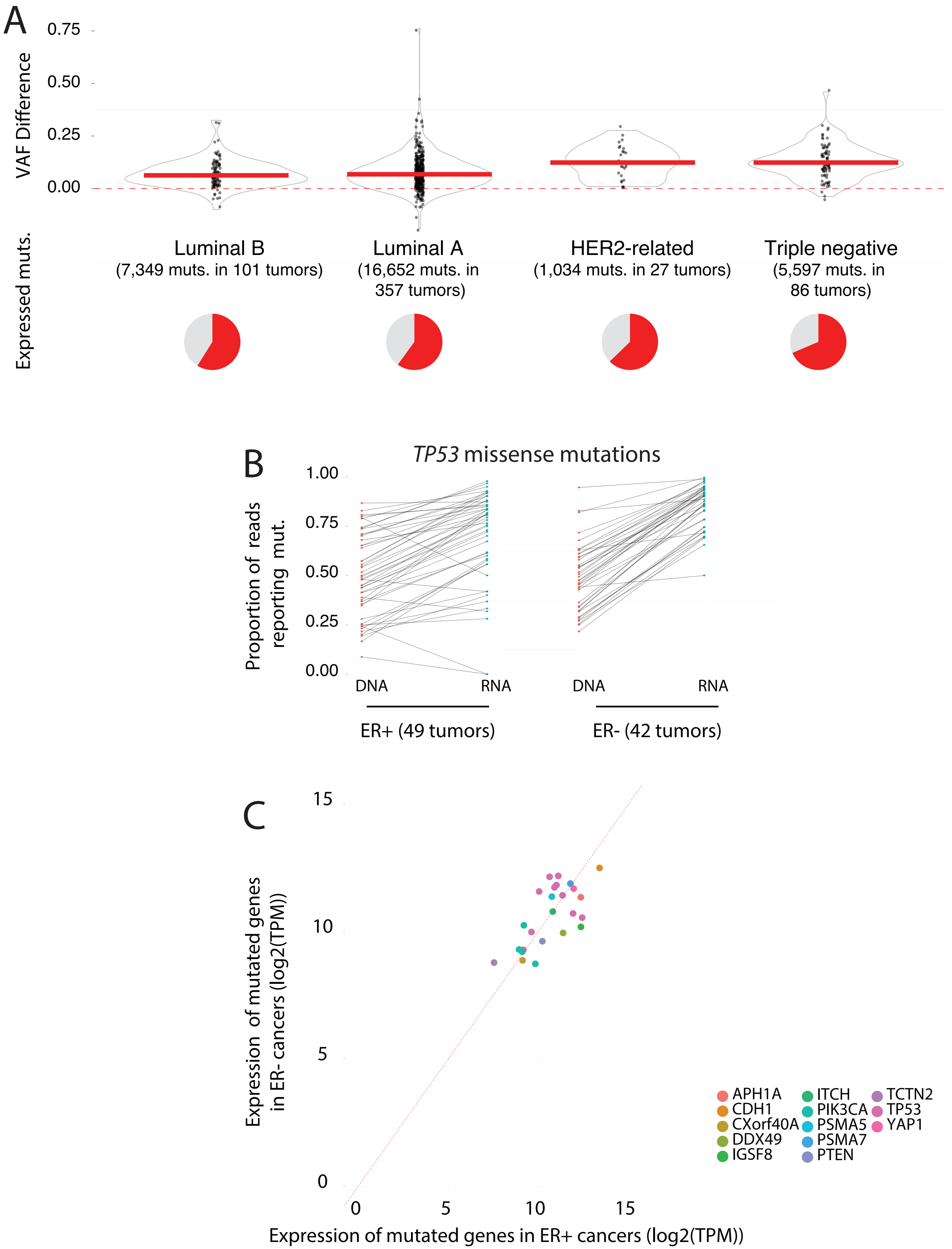

Supplementary figure S7
